# High crystallinity design of Ir-based catalysts drives catalytic reversibility for water electrolysis and fuel cells

**DOI:** 10.1038/s41467-021-24578-8

**Published:** 2021-07-13

**Authors:** Woong Hee Lee, Young-Jin Ko, Jung Hwan Kim, Chang Hyuck Choi, Keun Hwa Chae, Hansung Kim, Yun Jeong Hwang, Byoung Koun Min, Peter Strasser, Hyung-Suk Oh

**Affiliations:** 1 Clean Energy Research Center, Korea Institute of Science and Technology (KIST), Seoul, Republic of Korea; 2 Center for Electronic Materials, Korea Institute of Science and Technology (KIST), Seoul, Republic of Korea; 3 Department of Chemical and Biomolecular Engineering, Yonsei University, Seoul, Republic of Korea; 4 School of Materials Science and Engineering, Gwangju Institute of Science and Technology, Gwangju, Republic of Korea; 5 Advanced Analysis Center, Korea Institute of Science and Technology (KIST), Seoul, Republic of Korea; 6 Department of Chemistry, Seoul National University, Seoul, South Korea; 7 Center for Nanoparticle Research, Institute for Basic Science (IBS), Seoul, South Korea; 8 Graduate School of Energy and Environment (KU-KIST Green School), Korea University, Seoul, Republic of Korea; 9 The Electrochemical Energy, Catalysis, and Materials Science Laboratory, Department of Chemistry, Chemical Engineering Division, Technical University Berlin, Berlin, Germany; 10 Division of Energy and Environmental Technology, KIST School, Korea University of Science and Technology, Seoul, Republic of Korea; 11 KHU-KIST Department of Conversing Science and Technology, Kyung Hee University, Seoul, Republic of Korea

**Keywords:** Fuel cells, Electrocatalysis, Nanoscale materials

## Abstract

The voltage reversal of water electrolyzers and fuel cells induces a large positive potential on the hydrogen electrodes, followed by severe system degradation. Applying a reversible multifunctional electrocatalyst to the hydrogen electrode is a practical solution. Ir exhibits excellent catalytic activity for hydrogen evolution reactions (HER), and hydrogen oxidation reactions (HOR), yet irreversibly converts to amorphous IrO_x_ at potentials > 0.8 V/RHE, which is an excellent catalyst for oxygen evolution reactions (OER), yet a poor HER and HOR catalyst. Harnessing the multifunctional catalytic characteristics of Ir, here we design a unique Ir-based electrocatalyst with high crystallinity for OER, HER, and HOR. Under OER operation, the crystalline nanoparticle generates an atomically-thin IrO_x_ layer, which reversibly transforms into a metallic Ir at more cathodic potentials, restoring high activity for HER and HOR. Our analysis reveals that a metallic Ir subsurface under thin IrO_x_ layer can act as a catalytic substrate for the reduction of Ir ions, creating reversibility. Our work not only uncovers fundamental, uniquely reversible catalytic properties of nanoparticle catalysts, but also offers insights into nanocatalyst design.

## Abstract (web summary)

Reversible multifunctionality in electrocatalysts can allow voltage reversal during device operation. Here, authors design a crystalline Ir-based electrocatalyst with a thin reversible metallic-Ir/IrO_x_ layer that shows activity for O_2_ evolution, H_2_ evolution, and H_2_ oxidation.

## Introduction

In view of a clean and renewable society, there has been great interest in water electrolysis and fuel cells for producing and using hydrogen, which is considered a promising energy carrier^1,2^. Water electrolysis consists of a two-electrode reaction: a hydrogen evolution reaction (HERs) and an oxygen evolution reaction (OERs)^3,4^. Fuel cells produce electricity through two-electrode reactions: hydrogen oxidation (HOR) and oxygen reduction (ORR)^5^. Recent studies have reported that unexpected operating conditions, such as a sudden shut-down and fuel starvation, induce voltage reversal to corrode hydrogen electrodes, degrading the durability of the systems. When shut-down occurs in water electrolysis, the potential of the hydrogen electrode is increased, leading to degradation at the hydrogen electrode^6,7^. In polymer electrolyte membrane (PEM) fuel cell operation, fuel starvation occurring at the anode side leads to a voltage reversal phenomenon, causing corrosion of carbon components like catalyst support, gas diffusion layer (GDL) and flow field plate on the anode^8,9^. A recent study proposed reducing the damage to the electrode by selectively promoting HOR catalysts by suppressing the ORR^10^ and introducing a water oxidation catalyst to the anode of the PEM fuel cell to induce an OER, as it is a reaction that competes with the carbon corrosion reaction^9^. In this regard, multifunctional catalysts can be a promising strategy in environments where the electrochemical reaction changes rapidly, such as the voltage reversal of water electrolysis and PEM fuel cell systems.

Among the various catalysts, Ir-based materials are excellent candidates to fit this strategy owing to their remarkable OER activity as well as good HER and HOR catalytic activity^11–14^. In particular, an amorphous IrO_x_ surface is considered the best active site for OER^15,16^. Based on the X-ray photoemission spectroscopy (XPS) and X-ray absorption spectroscopy (XAS), amorphous IrO_x_ (Ir^III^) exhibits a higher oxidation state than rutile IrO_2_ (Ir^IV^) due to abundant electrophilic oxygen species (O^I‒^) which cause nucleophilic attack of water, leading to enhanced OER catalytic activity^17–19^. For HER and HOR, it has been reported that metallic-Ir surfaces such as Ir (111) show significant catalytic activities^20,21^. Thus, Ir-based materials possess good OER, HER and HOR catalytic activity and can be used as anodes and cathodes of water electrolyzers and as anodes of PEM fuel cells. However, despite the multifunctionality of Ir-based catalysts, the inconsistent active sites and irreversibility of Ir for HER, HOR, and OER have limitations in their application to real device systems with varying operating conditions. The Strasser group reported that Ir nanoparticles irreversibly changed to oxide formation under potential cycling over 1.0 V_RHE_^14^. Fuel starvation in PEM fuel cells or voltage reversal in water electrolysis occurs, and irreversible oxidation of Ir-based catalyst would happen at the anode in the fuel cell and at the cathode in water electrolyzers, resulting in the loss of multifunctional catalytic properties. Therefore, securing the reversible properties of Ir nanoparticles can be a viable alternative that can improve the durability of fuel cells and water electrolysis systems while maintaining multifunctional catalytic properties.

Herein, we introduce a crystalline Ir-based alloy catalyst as a strategy to maintain the electrochemical reversibility of Ir. Alloying Ir with a transition metal is an efficient method to improve the kinetics of the OER, HER and HOR^22,23^. We synthesize carbon-supported IrNi alloy nanoparticles with high crystallinity (IrNi/C-HT) and with low crystallinity (IrNi/C-LT), both of which exhibited excellent OER/HER/HOR catalytic activity. Based on the results of in situ*/operando* X-ray absorption near edge structure (XANES) and depth-resolved XPS, HER and HOR performance of IrNi/C-LT sharply decreased after the OER measurement, due to conversion of the irreversible amorphous IrNiO_x_ surface. However, IrNi/C-HT possesses a very thin IrNiO_x_ layer under the OER condition and this IrNiO_x_ layer is reversibly converted to a metallic surface under the HER condition, exhibiting high HER performance. We explored the mechanism of the reversible IrNiO_x_ layer of IrNi/C-HT using electrochemical flow-cell coupling *operando* inductively coupled plasma-mass spectrometry (ICP-MS). In addition, the prepared catalysts were applied to the fuel starvation of the PEM fuel cell and the reverse voltage of the water electrolyzer to confirm their viability in a real environment.

## Results

### IrNi/C morphology and crystallinity: synthesis temperature

**Fig. 1:**
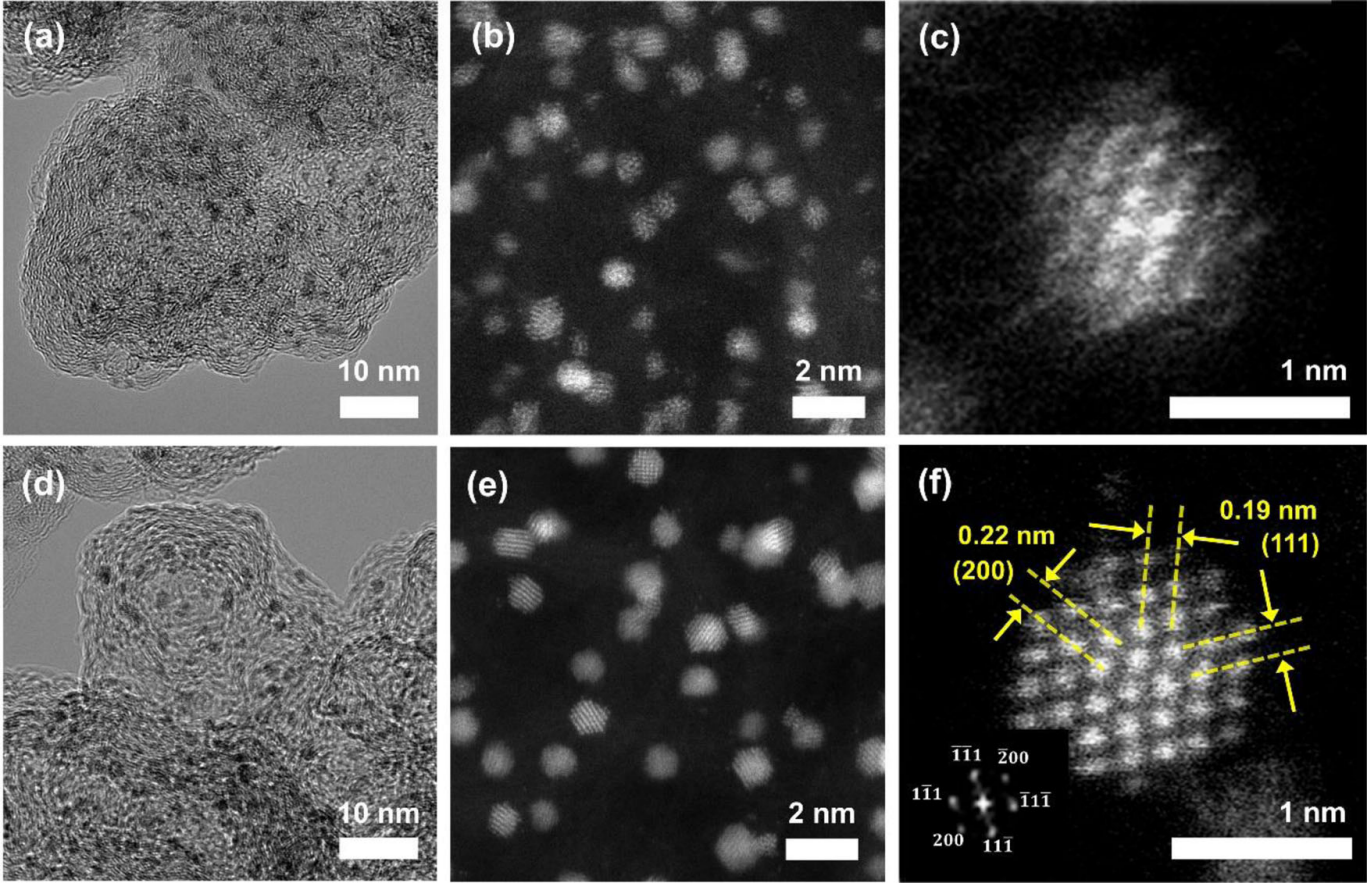
Morphological and structural characteristics of the synthesized IrNi nanoparticles supported on carbon at different heat-treatment temperatures of 400 °C and 1000 °C, which are denoted as IrNi/C-LT and IrNi/C-HT, respectively. **a** TEM and **b**, **c** HADDF images of IrNi/C-LT. **d** TEM and **e**, **f** HADDF images of IrNi/C-HT. The inserted fast Fourier transform of the IrNi/C-HT particles show the (111) and (200) planes.

IrNi nanoparticles supported on carbon (IrNi/C) were synthesized using a modified impregnation method that instantly changed to reductive gas conditions at target heat-treatment temperatures, to control the crystallinity while maintaining a small particle size. Carbon was used as the model support for observing nanocatalyst properties. Synthesized IrNi/C materials treated at 400 °C and 1000 °C were denoted as IrNi/C-LT and IrNi/C-HT, respectively. For comparison, Ir/C-LT and Ir/C-HT were prepared by the same method without the nickel precursor. The high-resolution transmission electron microscopy (HR-TEM) images of IrNi/C-LT and -HT (Fig. 1a, d) show a similar particle size (1–1.5 nm) and distribution. Energy-dispersive X-ray (EDX) mapping images of the IrNi/C catalysts (Supplementary Figs. 1–3) reveal that Ir and Ni elements are moderately distributed on the carbon support, suggesting the presence of an Ir and Ni alloy. Based on the TEM EDX images (Supplementary Fig. 4), the atomic ratios of Ir:Ni were 78:21 and 66:34 for IrNi/C-LT and IrNi/C-HT, respectively. However, as shown in the high-angle annular dark field (HADDF) image (Fig. 1b, c, e, f), IrNi/C-LT showed a polycrystalline and amorphous structure with low crystallinity, whereas IrNi/C-HT displayed an almost single crystal character with high crystallinity. The IrNi/C-HT nanoparticles exhibited (111) and (200) facets, suggesting [011] FCC single-crystal structure. The above crystallinity differences were confirmed by X-ray diffraction (XRD) (Supplementary Fig. 5). The (111) reflection of IrNi/C-HT was located at 41.1°, upshifted relative to that of metallic Ir at 40.6° (see JCPDS no. 87-0715), suggesting a disordered Ir-Ni alloy. In contrast, an Ir alloy peak was not observed for IrNi/C-LT due to its small particle size and low crystallinity. Sharp XRD reflections as well as HR-TEM images confirmed the presence of few large Ni particles (Supplementary Fig. 6). A similar relation between temperature and crystallinity at comparable particle size was observed for the pure Ir/C catalysts (Supplementary Figs. 7 and 8). Based on these results, we concluded that controlling the crystallinity of IrNi/C catalysts by the annealing temperature at comparable 1~2 nm particle size and alloy atomic ratios was achieved. The rational reason for the small particle sizes with high crystallinity as described above is as follows. First, the dry product of carbon support with Ir and Ni precursor containing solution leads to Ir and Ni ions attaching to the carbon support in atomic-scale fine dispersion. These are hardly reduced in N_2_ without H_2_ gas even at high temperatures. This prevents Ostwald ripening/agglomeration during thermal treatments of up to 1000 °C. Second, when the N_2_ gas was immediately changed to 10% H_2_ (99.999%) and 90% N_2_ at 1000 °C, reduction occurred quickly, leading to a higher nucleation density and a finer nuclei size. This effect can be inferred from conventional nucleation theory^24^. When the temperature is raised with an H_2_ atmosphere, the reduction proceeds slowly due to a low temperature. This leads to Ostwald ripening acceleration, resulting in a particle size that is not finer, as shown in Supplementary Fig. 9. In that state, only the IrNi/C-HT sample undergoes a sufficient crystallization step to form single-crystal nanoparticles with high crystallinity.

### Electrochemical properties and catalytic activity

**Fig. 2:**
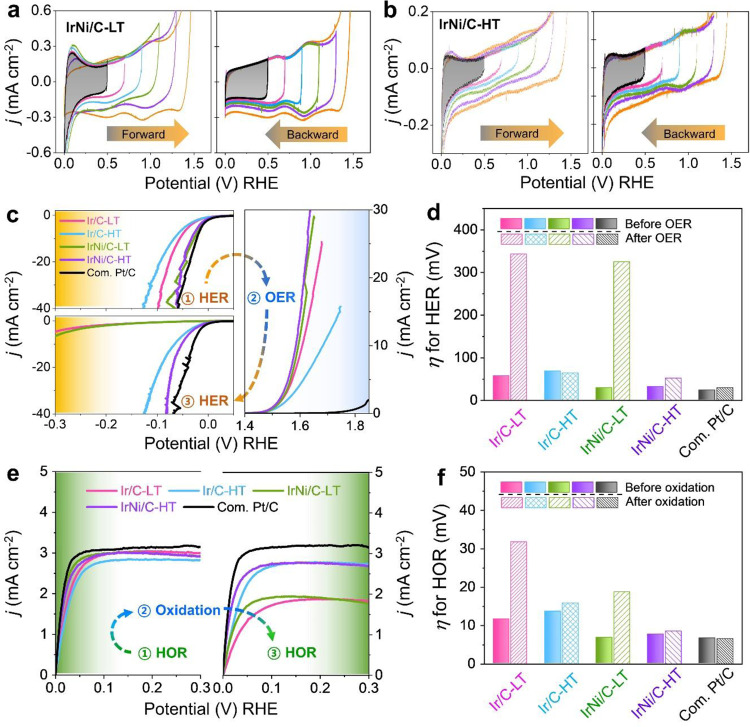
Electrochemical properties and catalytic reversibility of the Ir-based electrocatalyst. Cyclic voltammetry (CV) of **a** IrNi/C-LT and **b** IrNi/C-HT catalyst recorded with 50 mV s^–1^ in 0.05 M H_2_SO_4_ solution. The lower potential limit (LPL) is constantly 0.05 V_RHE_. The upper potential limit was increased from 0.5 to 1.5 V and decreased from 1.5 to 0.5 V in 200-mV steps. **c** iR-corrected current-potential curves for hydrogen evolution reaction (HER), oxygen evolution reaction (OER), and HER after OER. **d** Comparison between trends in activities for HER and HER after the OER test, expressed as overpotentials required for 10 mA cm^–2^. **e** iR-corrected current-potential curves for hydrogen oxidation reaction (HOR) and HOR after oxidation. Oxidation was conducted via five cycles of CV between 0.05 and 1.5 V. **f** Comparison between trends in activities for HOR and HOR after oxidation, expressed as overpotentials required for 1 mA cm^–2^.

To investigate the effect of crystallinity on the electrochemical properties, the cyclic voltammetry (CV) of the prepared IrNi/C-LT and IrNi/C-HT was measured under changing upper potential limits (UPLs), (Fig. 2a, b and Supplementary Fig. 10). As the UPL increased during the CV of IrNi/C-LT, the hydrogen adsorption-desorption (H_upd_) peak associated with a metallic Ir surface disappeared and two redox peaks associated with Ir(III)/Ir(IV) and Ir(IV)/Ir(>IV) emerged^25^. When the UPL was decreased, the CV shape of IrNi/C-LT was maintained. This indicated that the surface of the IrNi/C-LT irreversibly changed from a metallic-IrNi alloy to IrNiO_x_. In contrast, the H_upd_ peak area of IrNi/C-HT decreased with increasing UPL but was restored again as the UPL decreased. These findings suggested that the surface of IrNi/C-HT reversibly converted between a metallic character and an oxidic IrNiO_x_ character. Again, a similar behavior was obvious for pure Ir/C (Supplementary Fig. 11).

Figure 2c demonstrates the electrocatalytic performance of the catalysts during a set of subsequent linear voltammetric scans probing first the HER, then the OER, and then again the HER reactivity, all in 0.05 M H_2_SO_4_. Prior to the OER test, the catalysts were activated by 50 CV cycles. During this process, an IrO_x_ layer was generated and surface Ni was leached out. Compared with Ir/C catalysts, IrNi/C catalysts demonstrated higher HER catalytic activity, comparable with commercial Pt/C. Ir/C-LT demonstrated a more enhanced OER performance than Ir/C-HT, due to the large amount of redox active Ir centers represented by the redox charge (Fig. 2a, b). Ir-Ni alloys generally show superior catalytic activity for the OER, regardless of their crystallinity. To evaluate metal-oxide reversibility, HER performances were re-measured after the OER test. While the IrNi/C-HT and Ir/C-HT catalysts maintained high HER reactivity, that of the IrNi/C-LT and Ir/C-LT catalysts decreased. These distinctly different levels of reversibility are reflected by the HER overpotentials (Fig. 2d) and the Tafel plots (Supplementary Fig. 11), as well. Interestingly, analogous behavioral trends were observed in the HOR/OER reversibility (Fig. 2e, f): Initial HOR performance of IrNi/C catalysts is comparable with that of commercial Pt/C catalyst. After 10 CV cycles, the HOR catalytic activity of IrNi/C-LT and Ir/C-LT catalysts dropped markedly, but that of IrNi/C-HT and Ir/C-HT catalysts decreased only slightly. These results demonstrate that Ir-based nanocatalysts with high crystallinity show remarkable reversibility in their surface electrochemistry and their associated electrocatalytic reactivity for oxidation and reduction reactions, such as HER/OER and HOR/OER. To further study the catalytic reversibility of Ir-based catalysts, the HER tests were conducted for Ir/C and IrNi/C before and after OER by changing the heat-treatment temperature, Ni amount ratio, and H_2_SO_4_ electrolyte concentration (Supplementary Figs. 13~16). Catalytic reversibility was found both Ir/C and IrNi/C catalysts heat-treated above 800 °C and was not affected by alloying effect and electrolyte concentration. These results clearly reveal that reversibility is solely derived from the crystallinity of Ir not Ni alloy. The Ni element only increases the catalytic activity regardless of the crystallinity, so this reversibility study focused on the analysis of Ir materials.

### Operando surface electronic structure of IrNi/C catalysts

**Fig. 3:**
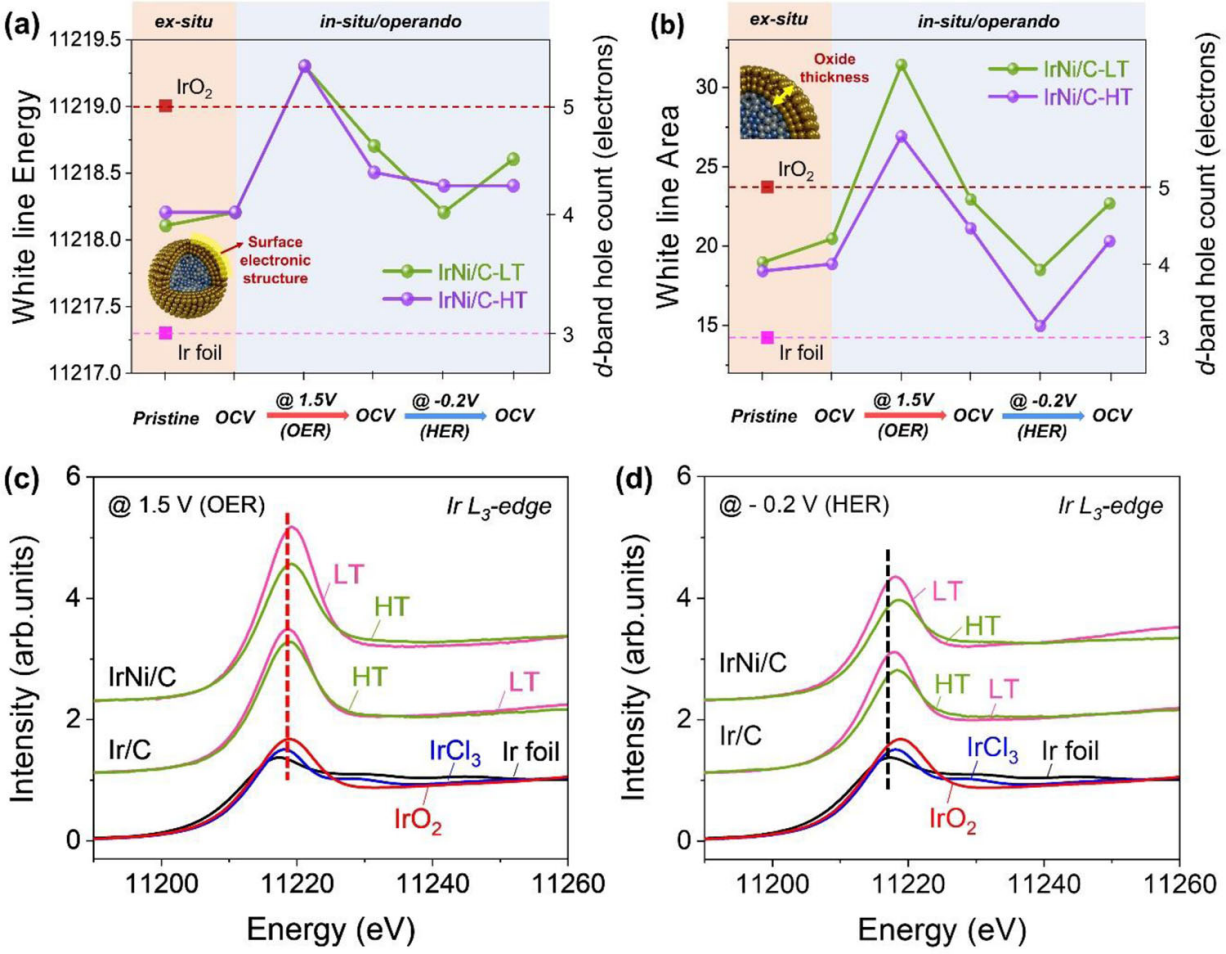
In situ*/operando* study of Ir-based electrocatalysts for electronic structure and oxide thickness analysis. Ir L3-edge XANES of synthesized catalysts measured under the experimental sequence: (i) pristine electrocatalysts powder, (ii) spray coated on electrode in 0.05 M H_2_SO_4_ at OCV, (iii) OER operating condition at 1.5 V_RHE_, (iv) OCV after OER operation, (v) HER operating condition at –0.2 V_RHE_, (vi) OCV after HER operation. (**a**) The white-line peak energy and (**b**) area of IrNi/C-LT and IrNi/C-HT are illustrated. Commercial Ir black and IrO_2_ were measured as references under same conditions. Ir *L*_*3*_-edge XANES spectra of synthetic catalyst during (**c**) OER and subsequent (**d**) HER conditions. XANES spectra under other experimental conditions are shown in Supplementary Figs. 18 and 19.

To explain the reversible character of the high crystallinity Ir-based catalyst, we tracked the electronic structure and oxide thickness of IrNi@IrNiO_x_ core-shell nanoparticles under intermittent OER and HER operating conditions using in situ*/operando* X-ray absorption near-edge structure (XANES) of Ir L_3_-edge to probe electron transitions from *2p* to *5d*. Since XANES is a bulk sensitivity technique, it is difficult to show the oxidation state of local structures. The white line of core-shell Ir nanoparticles represents the overall *d*-hole character, which is the redox state of both the oxidized Ir in the shell and that of the metallic Ir in the core. If the core of the catalyst state is fixed to metallic-Ir, any shift of the white-line peak energy of the core-shell catalyst roughly represents a change in the *d*-band hole count of Ir in the shell (Fig. 3a)^26^. Moreover, if the particle size is the same, the oxide thickness of the core-shell can be roughly estimated by the white-line area (Fig. 3b). A further detailed explanation is shown in Supplementary Note 5.

The white-line peak energy in the experimental sequence is illustrated in Fig. 3a. Under the OER condition, the white-line peak energy of both IrNi/C catalysts is positively shifted with a *d*-band hole count of 5.4 compared with the Ir/C catalyst (*d*-band hole count of 5.2), as shown in Fig. 3c. The *d*-band hole count was calculated by white-line peak energy and formal *d*-band hole count function (Supplementary Fig. 17). The large number of vacancies in IrNiO_x_/C enhances the electrophilic character of oxygen, resulting in smaller kinetic barriers for OER^26–28^. These XANES results can explain the high activity of IrNi/C catalysts for OER. After OER and OER-HER operations, IrNi/C-LT has a higher white-line peak energy than IrNi/C-HT, indicating a higher oxidation state of IrNi/C-LT after reaction. However, under HER condition, the white-line peak energy of IrNi/C-HT is higher than that of IrNi/C-LT (Fig. 3d). To explain this phenomenon, we estimated the chemical state of the catalyst under HER conditions. The HER Tafel slope of IrNi/C-HT (43 mV dec^–1^) indicates that the Heyrovsky step in the Volmer-Heyrovsky mechanism is the rate determining step for IrNi/C-HT^29^. The adsorbed hydrogen (H_ads_) coverage is expected to be 0.25–0.50 for the Heyrovsky step^30^. Based on previous research, H_ads_ increases the *d*-band hole count^31,32^. Thus, the H_ads_ coverage of IrNi/C-HT would slightly increase the white-line peak energy during the HER. For IrNi/C-LT, it is assumed that the IrO_x_ surface adsorbs protons at a cathodic potential and converts to an Ir(O_x_H_y_) species that possesses a lower *d*-band hole count than IrO_x_^33,34^.

To observe the oxide thickness under OER/HER operating conditions, white-line areas were identified by in situ*/operando* XANES. Under all conditions, IrNi/C-HT catalysts showed smaller white-line areas than IrNi/C-LT catalysts, implying that IrNi/C-HT possesses a thinner IrO_x_ layer than IrNi/C-LT during the OER and HER (Fig. 3b). In particular, the IrNi/C-HT catalyst showed a highly reduced white-line area under the HER condition (Fig. 3d), which is close to the metallic-Ir foil. Thus, IrNi/C-HT nanoparticles possess an almost metallic surface under the HER condition, leading to high HER activity. The white-line area clearly exhibits the difference between IrNi/C-HT and IrNi/C-LT under OER and HER condition. This suggests that oxide thickness is a key controlling factor of the reversibility phenomenon of IrNi@IrNiO_x_ core-shell nanoparticles with high crystallinity.

**Fig. 4:**
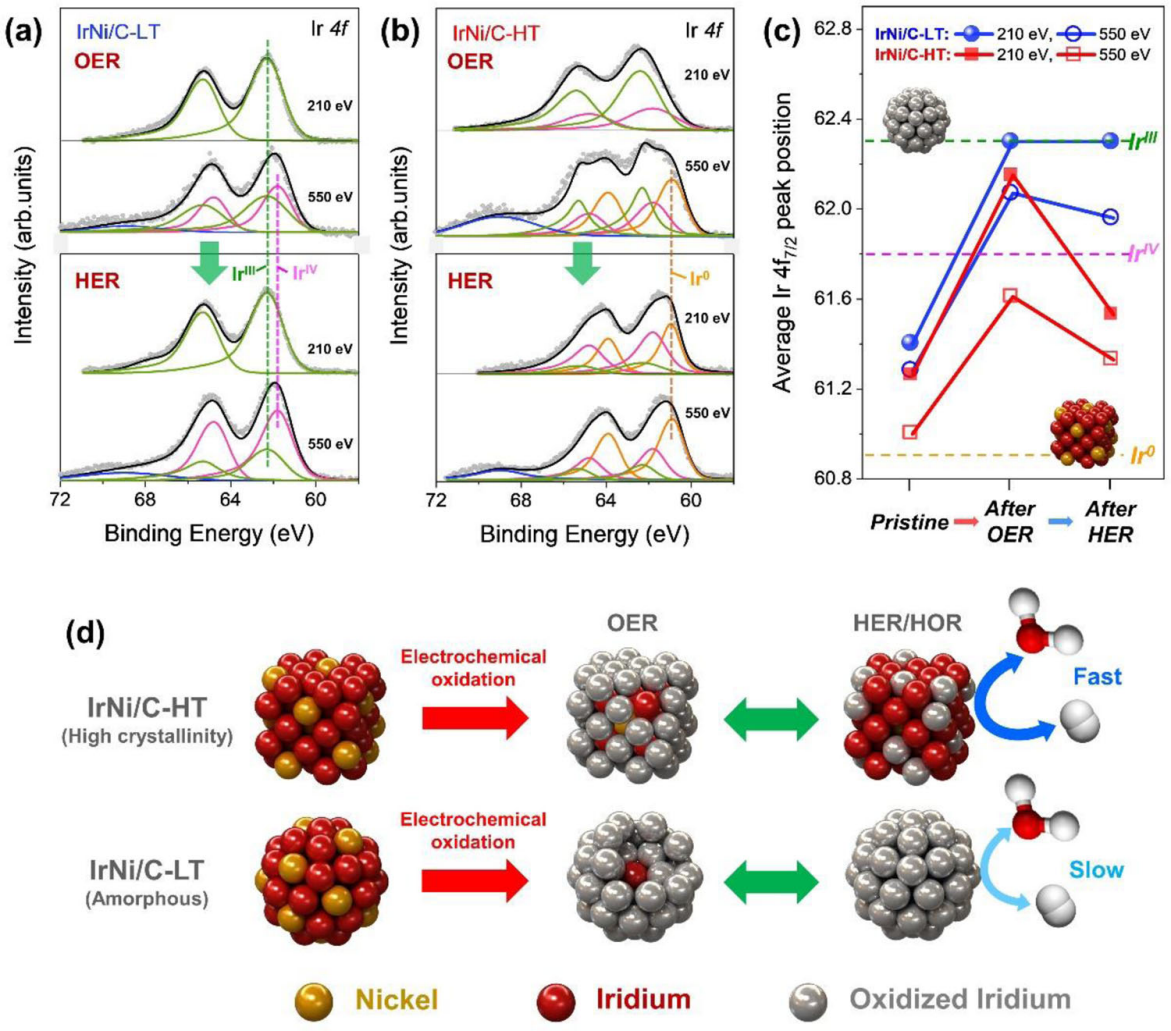
Surface electronic structure of Ir-based catalysts with different crystallinities. Depth-resolved Ir 4 f spectra of (**a**) IrNi/C-LT and **b** IrNi/C-HT after OER and followed by HER at photoelectron kinetic energies of 210 and 550 eV. **c** Average Ir *4* *f* peak position of synthesized catalyst under experimental sequence with different photoelectron kinetic energies of 210 and 550 eV. **d** Schematic illustration of IrNi/C-LT and IrNi/C-HT under OER and HER/HOR.

To study the effect of crystallinity on the surface electronic structure in more detail, we analyzed a depth-resolved Ir *4* *f* XPS spectra using two different levels of kinetic energy (KE). The associated inelastic mean free paths associated with KEs of 210 eV and 550 eV are almost 0.5 and 0.9 nm, respectively, which represent the atomic shell layers of 2 and 3–4 Ir atoms, respectively^35^. The Ir *4* *f* spectrum can be deconvoluted into the three types of Ir species: Ir^0^ (metallic-Ir), Ir^IV^ (rutile-type Ir^IV^O^II–^_2_), and Ir^III^ (amorphous Ir^III^O^I^_3_)^19^. The details of the deconvoluted XPS results are shown in Supplementary Table 1. The Ir depth profile of pristine IrNi/C electrodes indicates that the majority of nanoparticles were in the metallic state with soft oxidation of the surface (Supplementary Fig. 20). After OER, the spectrum of the IrNi/C-LT catalyst at 210 eV KE is fully shifted to the position of the 100% Ir^III^ species (Fig. 4a). The Ir 4 f line shape of the IrNi/C-LT catalyst at 550 eV KE is a mixture 53.2% Ir^III^ and 46.8% Ir^IV^ with no metallic species, suggesting that the surface of IrNi/C-LT is completely changed to a thick amorphous IrNiO_x_ layer. This thick amorphous IrNiO_x_ shell of IrNi/C-LT is conserved after the HER condition, revealing an irreversible Ir oxide layer on IrNi/C-LT. Meanwhile, the composition of the IrNi/C-HT catalyst after OER is 69.6% Ir^III^ and 30.4% Ir^IV^ at 210 eV KE and 39.4% Ir^III^, 26.8% Ir^IV^, and 39.4% Ir^0^ at 550 eV KE (Fig. 4b), indicating that an thin IrNiO_x_ layer has been synthesized on the metallic subsurface. After HER, the ratio of Ir^III^ species at 210 eV KE is significantly decreased and 38.2% metallic-Ir is observed, demonstrating that some of the thin IrNiO_x_ layer was turned into the metallic surface. The Ir depth profiles of IrNi/C-LT and IrNi/C-HT summarized in Fig. 4c, clearly demonstrate that the thick IrNiO_x_ layer of IrNi/C-LT synthesized by OER is maintained after HER and that the thin IrNiO_x_ layer of IrNi/C-HT converts to metallic-Ir after HER.

The description of phenomena related to crystallinity and HER/HOR/OER reversibility is illustrated in Fig. 4d. IrNi/C-LT with low crystallinity exhibits an abundant grain boundary, which accelerates the penetration of ions into nanoparticles and results in the formation of a thick IrNiO_x_ layer^36^. *Ex-situ* HAADF and EDS mapping images show that IrNi/C-LT (Supplementary Fig. 21) consists of a thick amorphous IrNiO_x_. This thick irreversible IrNiO_x_ demonstrates excellent performance for OER but poor performance for the HER and HOR. On the other hand, IrNi/C-HT with high crystallinity has no grain boundary defects, enabling formation of a very thin IrNiO_x_ layer. Ni of IrNi/C-HT was still located with Ir, confirming the core of the metallic structure (Supplementary Fig. 22). This thin IrNiO_x_ of IrNi/C-HT is reversibly converted to a metallic structure under HER/HOR conditions, showing high reversibility for OER/HER/HOR.

### Mechanistic study of reversible catalytic activity

**Fig. 5:**
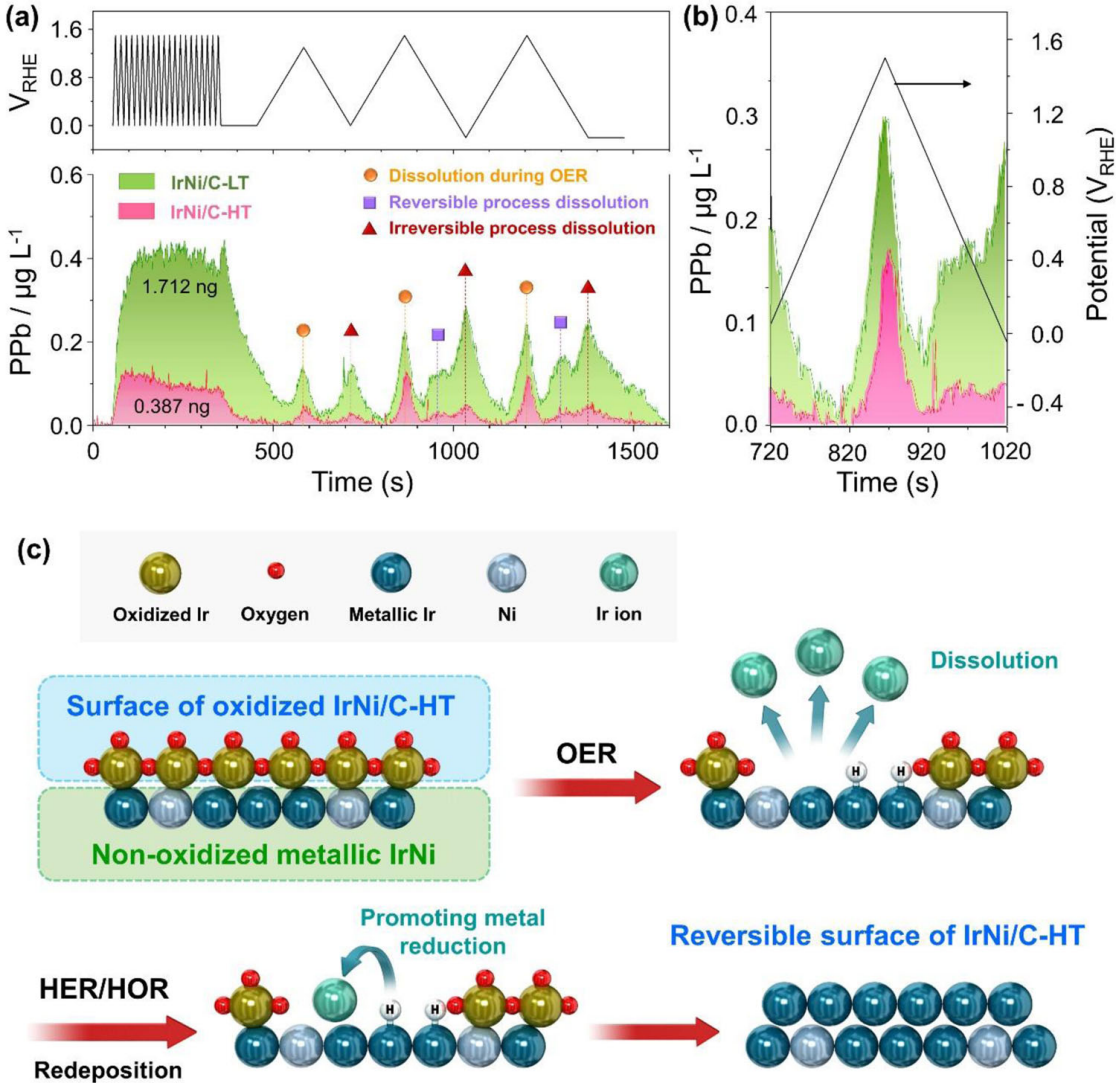
Mechanistic study of the reversible catalyst surface by in situ*/operando* ICP of IrNi/C-LT and IrNi/C-HT. **a** Real-time Ir dissolution profile of IrNi/C-LT and IrNi/C-HT under a representative experimental sequence. **b** The Ir dissolution profile during CV in the scan range between 0 and 1.5 V_RHE_. **c** Reversible surface schematic of IrNi/C-HT under OER- HER conditions.

To further clarify the reversible reactivity character of the high crystallinity Ir-based catalyst, we correlated it to surface dissolution processes at the atomic scale and were able to uncover the underlying mechanism that leads to reversibility. To achieve this, we conducted in situ*/operando* ICP technique using an electrochemical flow cell^37^. It is well known that electrochemically prepared IrO_x_ is irreversibly oxidized^38–41^. Hence, there is a need for a mechanistic study on the cause of reversible conversion of the IrNiO_x_ layer on IrNi/C-HT to a metallic surface. When cycling between 0.05 and 1.5 V_RHE_ to produce IrNiO_x_ species on nanoparticles, IrNi/C-LT has a large peak compared to IrNi/C-HT, indicating that IrNi/C-LT possesses a large amount of amorphous IrNiO_x_. Subsequently, three types of peaks were observed (Fig. 5a, b).

First, the peaks of OER intermediate dissolution located at the highest potential, are associated with an intermediate Ir species during the OER and lattice oxygen participation (denoted as O1 ~ O3). For the peak area of dissolution during the OER, IrNi/C-HT is lower than that of IrNi/C-LT owing to the thin IrNiO_x_ layer of IrNi/C-HT. To confirm stability of IrNi/C-HT during reaction, the dissolution profile under OER and HER conditions was obtained and is shown in Supplementary Fig. 23. IrNi/C-HT under OER and HER conditions showed a lower Ir dissolution than IrNi/C-LT, despite similar OER catalytic activity (Fig. 2c), demonstrating high stability of IrNi/C-HT. Second, the peaks of reversible reduction dissolution (denoted as R1 and R2) which are located close to 1.0 V_RHE,_ close to the standard potential of Ir oxidation reaction (0.926 V + 0.0591 pH), exhibited electrochemical dissolution of reversible Ir oxides. Third, the peaks of irreversible reduction dissolution (denoted as I1, I2, and I3) were located at the lowest potential, representing cathodic dissolution due to irreversible Ir oxide. Both peaks indicate the cathodic dissolution of Ir oxide. Interestingly, there is no peak of reversible reduction dissolution for IrNi/C-LT, and IrNi/C-HT after 1.3 V. When the upper potential was increased from 1.3 to 1.5 V_RHE_, a peak of reversible reduction dissolution was observed, indicating the production of reversible Ir oxides during the OER^40^. For IrNi/C-HT, cathodic dissolution is significantly lower than that of IrNi/C-LT, indicating that metallic surface of IrNi/C-HT is not derived by removal of IrNiO_x_ layer through dissolution.

Mayrhofer *et al*. analyzed the reduction of Ir hydroxide using the in situ*/operando* ICP technique and suggested that cathodic dissolution can be explained by dissolution and re-deposition phenomena and/or incomplete oxide reduction^38,39^. Based on this hypothesis, we proposed a description of the reversible oxide film of IrNi/C-HT, as shown in Fig. 5c. The particularly small amount of cathodic dissolution of IrNi/C-HT suggests that the converted IrNiO_x_ almost all transformed to the metallic phase, and did not dissolve into the electrolyte. IrNi/C-HT has a thin IrNiO_x_ layer, resulting in a metallic surface after dissolution of IrNiO_x_. The adsorbed hydrogen (H_ads_) on the metallic surface can serve to promote Ir reduction to metal^42–44^. Based on the Tafel slope, the H_ads_ coverage expected to be 0.25 ~ 0.50 during HER is sufficient for metal reductions. This metallic surface of IrNi/C-HT served as a substrate for the deposition of dissolved Ir ions to accelerate the reduction of Ir ions to metallic-Ir, which supports the reversible property of IrNi/C-HT. For IrNi/C-LT, the thick oxide layer is retained despite cathodic dissolution. The IrNi/C-LT surface may have an IrO_x_H_y_ phase under HER conditions, leading to re-deposition of dissolved Ir ions and non-complete reduction of the oxide. Markovic *et al*. reported that dissolution/re-deposition phenomena can be measured by the amount of dissolution with different sweep rates^45^. Fast scan rates prevent dissolved ion from diffusion layer to bulk electrolyte, leading to a lower amount of dissolved ion. Thus, a high dissolution amount ratio of slow/fast scan rate exhibits enhanced dissolution/re-deposition property. The dissolution amount ratio of slow/fast scan rate of IrNi/C-HT between 0 and 1.5 V_RHE_ is 0.310$(\frac{0.113{\rm{ng}}}{0.364{\rm{ng}}})$, which is higher than 0.233$(\frac{0.381{\rm{ng}}}{1.634{\rm{ng}}})$ for IrNi/C-LT (Fig. 5a, b). To further prove dissolution/re-deposition property of IrNi/C-HT, H_upd_ peak area recovery was observed after OER and HER with different rotation rate of RDE. As seen in the sweep rate experiment, dissolution/re-deposition phenomena were affected by dissolved ion concentration, indicating that a low rotation rate of RDE could also lead to enhanced dissolution/re-deposition properties. In Supplementary Fig. 24, the increase of H_upd_ peak area after HER is higher at lower rotation rate of RDE. These results indicate that the reversibility of high crystallinity catalysts was derived by a dissolution/re-deposition mechanism.

### Harnessing catalytic reversibility in a large-scale single PEM fuel cell

**Fig. 6:**
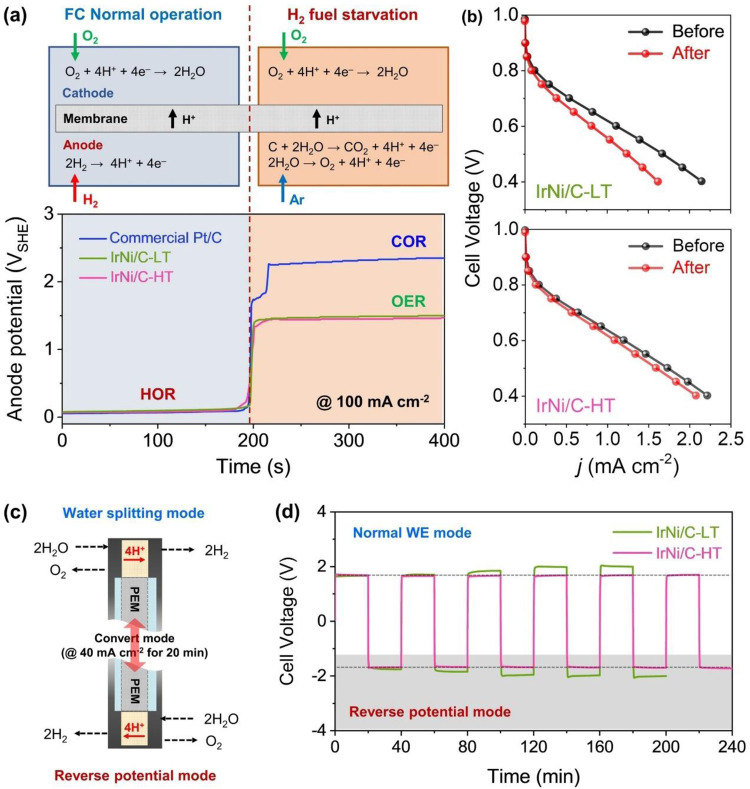
Reversibility of IrNi/C-HT in water electrolysis and fuel cell systems. **a** Scheme of the fuel starvation experiment at fuel cell and anode potential behaviors of the commercial TKK Pt/C, IrNi/C-LT and IrNi/C-HT as the anode catalysts under the fuel starvation condition. A single fuel cell test (geometric area: 5 cm^2^) employing commercial TKK 46% Pt/C as the cathode. **b** Polarization curves of IrNi/C-HT and -LT before and after fuel starvation experiment. **c** Scheme of the reverse voltage experiment in water electrolyzer. **d** Reverse voltage experiment using IrNi/C-LT and IrNi/C-HT as anode and cathode of a single water electrolyzer (geometric area: 10 cm^2^). Galvanostatic curves obtained with current density of 40 mA cm^‒2^.

To apply and harness the catalytic reversibility of Ir based nanoparticles with respect to the HOR and OER in a real electrochemical device, fuel starvation experiments were conducted in a single PEM fuel cell that was built using IrNi/C-HT and IrNi/C-LT as the catalytically active component in the anode catalyst layer (Fig. 6a). The initial fuel cell performance of IrNi/C-LT and -HT is lower than that of the commercial Pt/C catalyst due to the lower HOR activity and metal composition (Supplementary Fig. 25). For the fuel starvation experiment, the supplied anode gas was converted from H_2_ to Ar at a current density of 100 mA cm^‒2^. When H_2_ was exhausted at the anode, the anode potential of the commercial Pt/C catalyst increased to 1.8 V_RHE_ for OER instead of HOR, leading to a reverse potential phenomenon. After 20 s, the anode potential once more increased to 2.4 V_RHE_ for carbon oxidation reaction (COR), as shown in Fig. 6a and Supplementary Fig. 26^9^. This reaction conversion was confirmed by *in-situ* exhaust gas analysis (Supplementary Fig. 27). In contrast, the cell voltage of both IrNi/C cells increased and was maintained at 1.4–1.5 V for OER, and no CO_2_ gas was observed by in situ exhaust gas analysis, indicating that the high OER activity of IrNi/C catalysts prohibits corrosion of the carbon support. To observe HOR catalytic activity after fuel starvation, the fuel cell performance and impedance before and after the fuel starvation test were measured and are shown in Fig. 6b, Supplementary Figs. 25, 28, and Supplementary Table 2. The performance degradation at 0.6 V of commercial TKK Pt/C catalysts was 37.2% due to the carbon support corrosion. IrNi/C-LT reduced fuel cell performance by 26.9% despite no carbon corrosion, indicating low reversibility for HOR and OER. Conversely, the performance of IrNi/C-HT decreased slightly to 9.2%, suggesting that the reversible catalytic activity of IrNi/C-HT works well under real fuel starvation conditions.

To confirm the reversibility of IrNi/C-LT and -HT for HER and OER under real device conditions, reverse voltage experiments were performed in a water electrolyzer, with the cell polarity changed every 20 min at a constant current density of 40 mA cm^–2^ (Fig. 6c). As shown in Fig. 6d, both catalysts were applied with the same cell voltage of 1.65 V to obtain 40 mA cm^–2^ for the first 20 min. The cell voltage of IrNi/C-LT gradually increased whenever the polarity of the electrode was changed, but in the case of IrNi/C-HT, the cell voltage remained stable even after 10 polarity conversions. These results demonstrate that IrNi/C-HT retains bifunctional catalytic activity with reversibility to HER and OER under real device conditions. Our approach promotes the reversibility of nanocatalysts that enable a variety of electrochemical reactions, which can be used as catalysts to resist the reverse voltage in fuel cells and water electrolysis systems.

## Discussion

We designed a crystalline, multifunctional Ir based nano-catalyst with a remarkable reversibility in reactivity during intermittent switching between cathodic and anodic processes, such as HER/OER/HER. We studied and clarified the underlying mechanism of the reversibility at the molecular scale using a number of different in situ*/operando* analysis techniques. The key to the reversibility of crystalline Ir alloy nanoparticles is the thin IrNiO_x_ layers that form under OER condition. Based on the results of in situ*/operando* XANES and depth-resolving XPS profiles, thin IrNiO_x_ layers of IrNi/C-HT possess an increase in the number of *d*-band holes during OER, leading to excellent OER catalytic activity. Under HER conditions, this thin IrO_x_ layer was reversibly converted to metallic surface, which exhibited high catalytic activity for HER. As a result, IrNi/C-HT exhibits reversible catalytic activity for OER/HER/HOR. Our in situ*/operando* ICP results demonstrate that the reversible IrNiO_x_ layers emerge from a dissolution and re-deposition mechanism. The metallic IrNi alloy under the thin IrO_x_ layer of IrNi/C-HT can be a catalytic substrate for the reduction-dissolved Ir ions under HER conditions. Based on our reverse voltage experiment in a water electrolysis cell and our fuel starvation experiment at the PEM fuel cell, the IrNi/C-HT catalyst exhibits high reversibility for HER/OER and HOR/OER under real device conditions. Our nanocatalyst design with high crystallinity can impart unique reversible properties to provide insight into various catalytic reactions and applications.

## Methods

### Preparation of electrocatalysts

The IrNi nanoparticles supported on carbon (denoted as IrNi/C) electrocatalysts were prepared through a modified impregnation method. Sixty milligrams of iridium nitrate (Alfa Aesar), 45 mg of nickel acetate (Sigma Aldrich) and 260 mg of carbon black (Ketjen Black EC-300J) were ultrasonically mixed in 20 mL of deionized (DI) water. The mixed solution was dried under vacuum at 80 °C. The resultant powder was subjected to heat treatment under 100% N_2_ (99.999%) at a flow rate of 500 cm^3^/min to 400 °C, and then the gas was immediately changed to 10% H_2_ (99.999%) and 90% N_2_. The temperature was maintained at 400 °C for 5 min under the changed gas conditions and then cooled to room temperature. The resulting powder was washed 3 times with DI water and then filtered and dried in a vacuum oven at 40 °C for 12 h. The final product was denoted as IrNi/C-LT. Crystallized IrNi supported on a carbon catalyst was prepared by only varying the heat-treatment temperature to 1000 °C, and this was denoted as IrNi/C-HT. For comparison, Ir nanoparticles supported on carbon electrocatalysts synthesized at heat treatment temperatures of 400 °C and 1000 °C were prepared, which are denoted as Ir/C-LT and Ir/C-HT, respectively.

### Materials characterization

X-ray diffraction (XRD, PANalytical, Cu Kα radiation) was conducted to measure the crystallinity and identify the nature of the electrocatalysts. The morphology of the synthesized catalyst was measured by high-resolution transmission electron microscopy (HR-TEM) using an FEI Titan™ 80–300 TEM and an FEI Talos F200X. High-resolution scanning transmission electron microscopy (STEM) was performed by a Titan 80–300, and energy dispersive X-ray spectroscopy (EDX) was conducted by a Talos F200X. The elemental composition of the catalyst was analyzed using inductively coupled plasma-optical emission spectrometry (ICP-OES, iCAP 7000, Thermo Fisher Scientific). The depth profile of the Ir nanoparticles was characterized using depth-resolved X-ray photoelectron spectroscopy (XPS) at the 4-D beamline of Pohang Accelerator Laboratory (PAL), Pohang, South Korea. For the XPS measurements, samples were prepared by spraying and drying the catalyst ink onto carbon paper (Sigracet 39 BC) to prepare the electrode. After the OER, the electrodes were electrochemically treated in two steps. First, a cyclic voltammogram (CV) between 0.05 and 1.5 V_RHE_ was performed to oxidize the electrode for 50 cycles at a scan rate of 500 mV s^‒1^. Second, the OER proceeded for 10 min under a chronopotentiometry of 1.5 V_RHE_. For the electrodes after HER, the previous OER-treated electrodes underwent additional chronoamperometry at ‒0.2 V_RHE_ for 10 min.

### Electrochemical analysis

Electrochemical measurements were conducted on a VSP potentiostat (Bio-Logic) in a standard three-electrode system using a rotating disk electrode (RDE, 0.196 cm^2^, glassy carbon, Pine Instrument) as the working electrode and a Hg/Hg_2_SO_4_ electrode as the reference electrode. The electrochemical tests were performed in a 0.05 M H_2_SO_4_ aqueous solution. For the counter electrode, Pt wire was used for the OER and HOR, and a graphite rod was used for the HER. The measured potential was converted to a reversible hydrogen electrode (RHE) using calibration data, which were based on the CV result under H_2_ saturated 0.05 M H_2_SO_4_. For the RDE test, 5 mg of catalyst was dispersed in 2.49 ml of isopropyl alcohol (IPA), 2.49 ml of DI water, and 0.02 ml of 5 wt% Nafion^TM^ solution (Sigma) by sonication for 20 min. The mixed solution was loaded onto the RDE. The total Ir loading was fixed at 3 μg cm^‒2^. Prior to the OER test, the surface of the synthesized catalyst was oxidized using cyclic voltammetry (CV) between 0.05 and 1.5 V_RHE_ with 50 cycles at a scan rate of 500 mV s^‒1^. Ohmic resistance was measured by electrochemical impedance spectroscopy from 1000 to 0.1 Hz to compensate for iR loss.

### Reverse voltage test in a water electrolyzer

The water electrolysis test was conducted using a 10 cm^2^ single-cell water electrolyzer with two symmetric electrodes as both the anode and cathode. To fabricate the electrodes, the synthesized catalyst was ultrasonically mixed with 5 wt% Nafion^TM^ ionomer in IPA for 20 min. The prepared catalyst ink was directly sprayed onto a gold-coated Ti foam (Bekaert, 2GDL12N-50) at 90 °C using a heated vacuum table. The geometric area of the electrode was 10 cm^2^, and Ir at 0.1 mg cm^‒2^ was loaded onto the electrode. The prepared electrode was used simultaneously as an anode and a cathode and was separated by a Nafion 212 membrane. The prepared membrane electrode assemblies (MEAs) were sandwiched between the current collectors and assembled in a homemade cell body. The polarization curves were measured at room temperature and atmospheric pressure. DI water was pumped to the anode and cathode sides using a peristaltic pump. For the reverse voltage experiment, 40 mA cm^‒2^ was applied to the single cell, and the polarity of the electrode was reversed every 20 min. The details of the experiment are described in Supplementary Information.

### Fuel starvation test in a PEM fuel cell

The membrane electrode assemblies (MEAs) were prepared using the catalyst-coated membrane method. To fabricate the MEA, the commercial Tanaka Kikinzoku Kogyo (TKK) 46% Pt/C catalyst was used as the cathode, and the synthesized catalysts were employed as the anode. The catalysts were ultrasonically mixed with a Nafion® solution in IPA for 20 min and sprayed directly onto a Nafion 212 membrane with a 5 cm^2^ geometric area at 90 °C using a heated vacuum table. The catalyst-coated membrane was then placed in a hot press at 140 °C for 3 min. Pt loading at the cathode was 0.3 mg cm^‒2^, and noble metal loading at the anode was 0.1 mg cm^‒2^. Two Teflon gaskets were used to hold the MEA, and two gas diffusion layer (GDL) papers (Sigracet SGL 39BC) were used to diffuse the supplied gases (O_2_, H_2_, and Ar). The prepared gasket, GDL and MEA were stacked and applied to a single-cell polymer electrolyte membrane fuel cell (PEMFC).

The PEM fuel cell test was conducted at 75 °C at 1 atm with fully humidified O_2_ at the cathode and fully humidified H_2_ at the anode. The flow rate of each gas was 150 cm^3^/min. For the fuel starvation experiment, the current density was applied at 100 mA cm^‒2^, and the anode gas was changed from H_2_ to Ar to create fuel starvation conditions. The details of the experiment are described in the Supporting Information. The polarization curve and impedance were measured before and after the fuel starvation experiment. The exhausted gas at the anode during the fuel starvation experiment was analyzed using online gas chromatography mass spectrometry (GC-MS). The potential behavior under fuel starvation conditions was measured using a hydrogen reference electrode^9^.

### In situ*/operando* Ir L_3_-edge XANES

In situ*/operando* XANES measurements of Ir L_3_-edges were performed in fluorescence mode at the 1D beamline of Pohang Accelerator Laboratory (PAL), Pohang, South Korea. The XANES was measured using a custom-made electrochemical flow cell^37^. A Pt wire and graphite rod were used as counter electrodes for the OER/HOR and HER, respectively. For the reference electrode, Ag/AgCl (3 M NaCl) was used. The working electrode was prepared by spraying catalyst ink, which is used for MEA, on a carbon-coated Kapton film. The XANES spectra were collected at an angle of 45° with respect to the detector and beam line. The electrochemical measurements were performed in a 0.05 M H_2_SO_4_ aqueous solution. Peristaltic pumps were used to flow the electrolytes at 2 ml min^‒1^. Before the measurement of in situ*/operando* XANES during the OER, the electrode was electrochemically oxidized by CV between 0.05 and 1.5 V_RHE_ at a scan rate of 500 mV s^‒1^ for 50 cycles. After oxidation, XAS was measured at 1.5 V_RHE_ for in situ OER and ‒0.2 V_RHE_ for in situ HER. At each step, the catalyst was stabilized for 5 min before the XAS test. The acquired XANES and EXAFS data were processed using the ATHENA program. The details of the experiment are described in the Supplementary Information.

### In situ*/operando* ICP-MS measurements

In situ*/operando* ICP-MS tests were conducted using a custom-made electrochemical flow cell containing a three-electrode system^37^. An Ag/AgCl electrode (ALS Co., RE-3VT, 3 M NaCl) and graphite rod were used as the reference and counter electrodes, respectively. Catalyst ink was loaded onto the glassy carbon plate as a working electrode by drop casting a total area of 0.1 cm^2^. During the in situ*/operando* ICP-MS test, 0.05 M H_2_SO_4_ electrolyte was pumped at 0.8 ml min^‒1^ to the flow cell, which was also connected to an inductively coupled plasma mass spectrometer (ICP-MS). Re (1 ppb) was used as an internal standard to calibrate the Ir concentration. The details of the experiment are described in the Supplementary Information.

## Supplementary information

SUPPLEMENTARY INFORMATION

## Data Availability

The authors declare that the data supporting the findings of this study are available within the article and its Supplementary Information files. Additional data are available from the corresponding authors upon reasonable request.
