# Supplementary material for: High crystallinity design of Ir-based catalysts drives catalytic reversibility for water electrolysis and fuel cells
Source: Nat Commun. 2021 Jul 13;12:4271. doi: 10.1038/s41467-021-24578-8 (PMC8277764; doi:10.1038/s41467-021-24578-8)
Supplement: Supplementary file 1 — SUPPLEMENTARY INFORMATION [file 41467_2021_24578_MOESM1_ESM.pdf]

Supplementary Information

High crystallinity design of Ir-based catalysts drives catalytic reversibility for water electrolysis and fuel cells

Woong Hee Lee [1], Young-Jin Ko [2], Jung Hwan Kim [3], Chang Hyuck Choi [4], Keun Hwa Chae [5], Hansung Kim [3], Yun Jeong Hwang [6, 7], Byoung Koun Min [1, 8], Peter Strasser [9] *, and Hyung-Suk Oh [1, 10, 11] *

[1] Clean Energy Research Center, Korea Institute of Science and Technology (KIST), Hwarang-ro 14-gil 5, Seongbuk-gu, Seoul 02792, Republic of Korea

[2] Center for Electronic Materials, Korea Institute of Science and Technology (KIST), Hwarang-ro 14-gil 5, Seongbuk-gu, Seoul 02792, Republic of Korea

[3] Department of Chemical and Biomolecular Engineering, Yonsei University, 50 Yonsei-ro, Seodaemun-gu, 03722, Seoul, Republic of Korea

[4] School of Materials Science and Engineering, Gwangju Institute of Science and Technology, 123 Cheomdan-gwangi-ro, Buk-gu, Gwangju 61005, Republic of Korea

[5] Advanced Analysis Center, Korea Institute of Science and Technology (KIST), Hwarang-ro 14-gil 5, Seongbuk-gu, Seoul 02792, Republic of Korea

[6] Department of Chemistry, Seoul National University, Seoul 08826, South Korea

[7] Center for Nanoparticle Research, Institute for Basic Science (IBS), Seoul 08826, South Korea

[8] Graduate School of Energy and Environment (KU-KIST Green School), Korea University, 145 Anam-ro, Seongbuk-gu, Seoul 02841, Republic of Korea

[9] The Electrochemical Energy, Catalysis, and Materials Science Laboratory, Department of Chemistry, Chemical Engineering Division, Technical University Berlin, Berlin 10623, Germany

[10] Division of Energy and Environmental Technology, KIST school, Korea University of Science and Technology, Seoul 02792, Republic of Korea

[11] KHU-KIST Department of Conversing Science and Technology, Kyung Hee University, Seoul 02447, Republic of Korea

* Corresponding authors:
(P. Strasser) pstrasser@tu-berlin.de, (H. -S. Oh) hyung-suk.oh@kist.re.kr

Table of Contents

Contents	Page
○ Supplementary Note 1 …………………………………………………………	S3
> Reversal voltage experiment in water electrolyzer	
○ Supplementary Note 2 …………………………………………………………	S4
> Fuel Starvation experiment in PEM fuel cell	
○ Supplementary Note 3 …………………………………………………………	S5
> *In situ/operando* X-ray absorption spectroscopy (XAS)	
○ Supplementary Note 4 …………………………………………………………	S6
> *In situ/operando* ICP-MS	
○ Supplementary Note 5 …………………………………………………………	S7
> XANES analysis explanation for oxide thickness and oxidation state	
○ Supplementary Figures …………………………………………………………	S9
□ Supplementary Figures 1–28	
○ Supplementary Tables …………………………………………………………	S37
□ Supplementary Tables 1–2	

Supplementary Note 1

Voltage reversal test in water electrolyzer

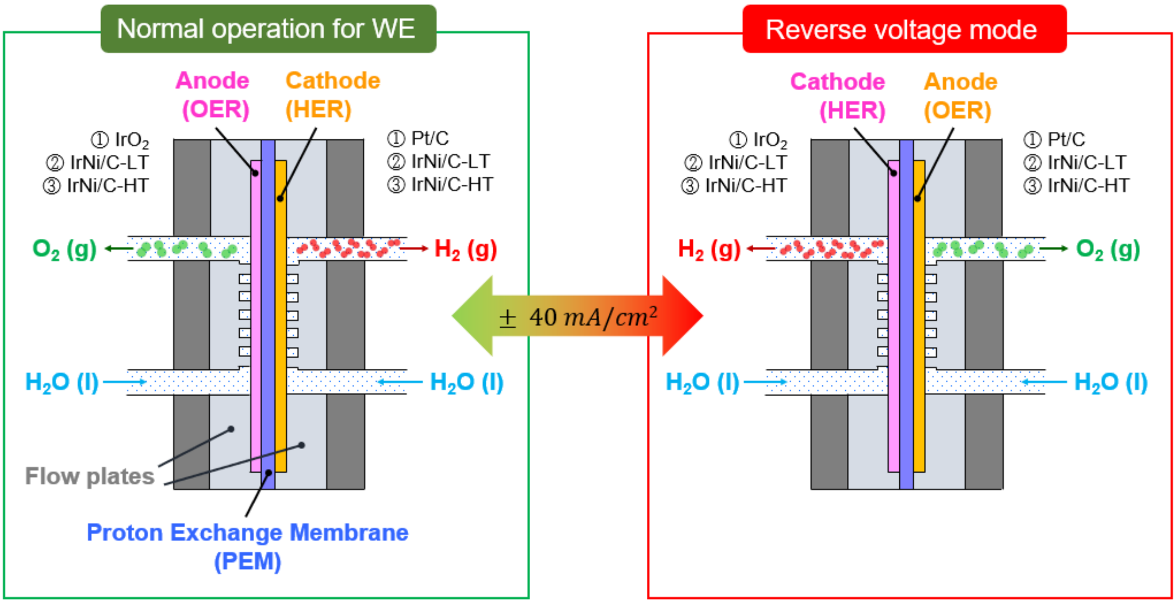

For the voltage reversal test, a round type 10cm^2 water electrolysis single cell was prepared. The diffusion layer (DL) was prepared by coating Au on Ti foam in order to exclude the influence of carbon corrosion. The synthesized IrNi/C-LT and -HT catalysts were applied to the diffusion layer (DL) and used as the anode and cathode. For comparison, commercial IrO_2 and TKK 46% Pt/C were used to the anode and cathode, respectively. DI water was supplied to both electrodes, and a current density of $\pm$40 mA cm^{-2} was applied every 20 min to observe the change in cell voltage. The single cell water electrolyzer was operated at room temperature without back pressure.

Supplementary Note 2

Fuel Starvation experiment in PEM fuel cell

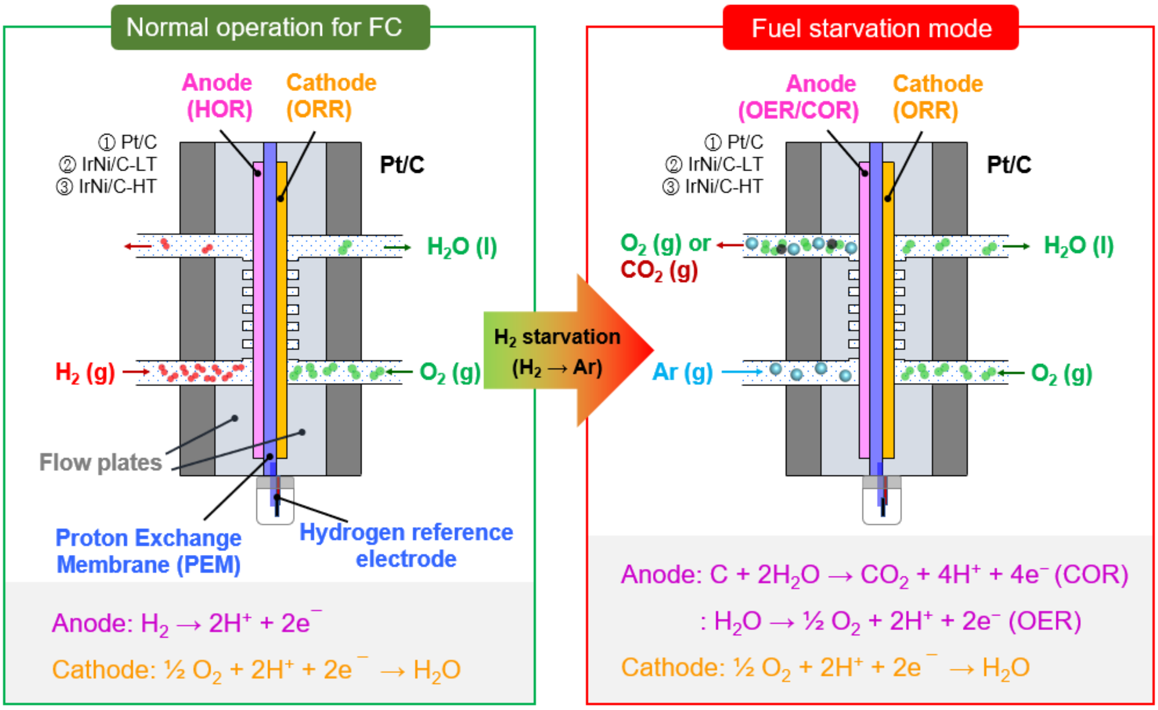

The fuel starvation test was conducted through a chronopotentiometry in which a constant current density of 100 mA cm^{-2} was applied while supplying 150 ccm of gas resource, such as H_2, O_2 and Ar, to the anode and cathode. First, the experiment is conducted under normal operating conditions in which H_2 and O_2 are supplied to the anode and cathode, respectively. And to form fuel starvation conditions, H_2, a source of protons and electrons, is replaced with inert Ar gas. The current density of 100 mA cm^{-2} was maintained for 600 s. During the fuel starvation experiment, the outline of anode was connected to online mass spectrometry to measure the amount of O_2 and CO_2. The cell potential was measured by VSP potentiostat (Bio-Logic). To measure anode potential behavior during fuel starvation phenomenon, a hydrogen reference electrode was connected to edge of MEA. The hydrogen reference electrode was prepared by following method. The Pt catalysts sprayed carbon paper was hot pressed onto end of the 1 cm × 10 cm Nafion 212 membrane at 140 °C for 5 min. The Pt wire was connected to carbon paper as a current collector. The manufactured electrode was connected to edge of MEA and the reference electrode was sealed with gas chamber. The gas chamber maintains H_2 condition by flowing H_2 gas and Nafion membrane was covered by moist Kimwipes® to maintain ionic conductivity of Nafion membrane.

Supplementary Note 3

***In situ/operando* X-ray absorption spectroscopy (XAS)**

Setup of *in situ/operando* XAS analysis with the homemade electrochemical flow cell is reported in previous paper.[1] The Ir-based electrocatalysts were deposited on the surface of the thin carbon film and contacted with 0.05 M H_2SO_4 electrolyte, which is a working electrode. Kapton film was applied on the back side of the working electrode facing the incident X-ray to avoid air entering the flow cell. The electrolyte is supplied below the flow cell by the peristaltic pump. Graphite rod and Ag/AgCl were used as the counter electrode and reference electrode, respectively, and were installed on the back side of the flow cell.

Supplementary Note 4

***In situ/operando* ICP-MS**

Setup of *in situ/operando* ICP−MS analysis using the homemade electrochemical flow cell having thin microchannel is reported in previous paper.[1] The IrNi/C electrocatalysts were loaded on a glassy carbon as a working electrode by drop-casting method. 0.05 M H_2SO_4 as an electrolyte was constantly flowed to the homemade electrochemical flow cell part along the microchannel by the peristaltic pump. The 1 M HNO_3 electrolyte with 1 ppb Re as an internal standard was constantly supplied end of the electrochemical flow cell to calibrate the experiment results. Graphite rod and Ag/AgCl (3 M NaCl) were used as the counter electrode and reference electrode, respectively, and were installed on the flow cell.

Supplementary Note 5

XANES analysis explanation for oxide thickness and oxidation state

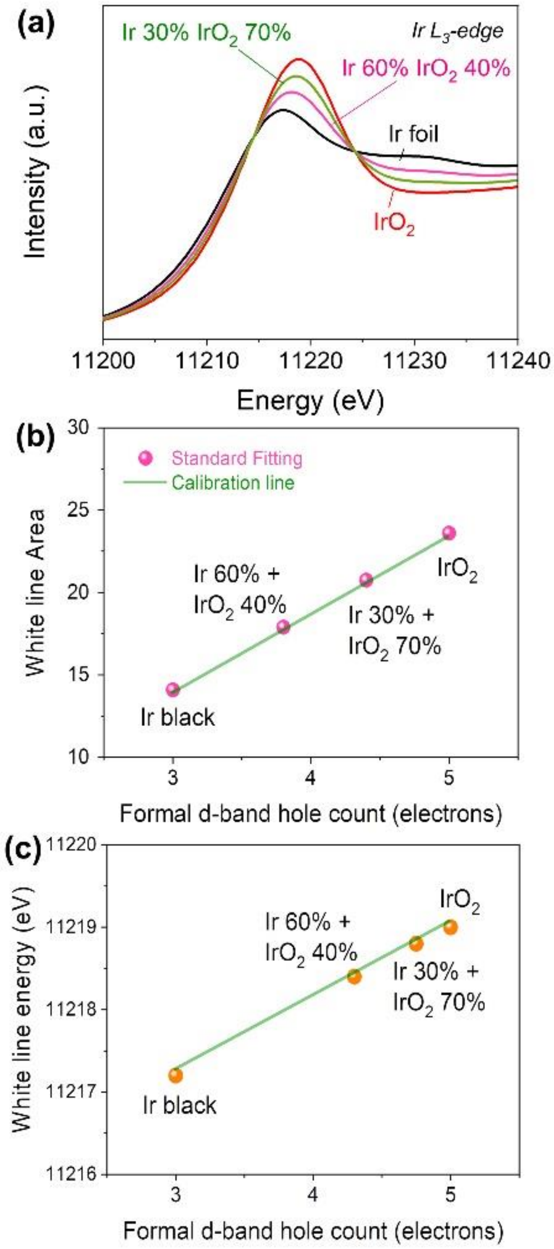

Supplementary Note 5 Figure. (a) XANES, (b) white line area, and (c) white line peak energy of varying the ratio of pure IrO_2 and fully reduced metallic-Ir with similar particle size.

The white line peak energy and peak area of Ir-based electrocatalysts are proportional with occupancy of the Ir 5d orbitals. These two information generally show the same trend when the Ir-based catalyst has one electronic structure, such as pure IrO_2 and fully reduced metallic-Ir. If the particle size is the same, as the oxidation state increases, the intensity and area of XANES peak increase and have a high energy position. However, if the Ir-based

catalyst has a mixed electronic structure, the white line peak energy and XANES peak area show slightly different information and trends. XANES is a bulk sensitivity technique, and their mixed electronic structure is expressed as the sum of each electronic structure information. These properties can be analyzed in detail through linear combination fitting (LCF).

For the white line area of mixture, it is same with the sum of the white line area of each element. However, white line peak energy of mixture is not same with the average of white line peak energy of each element. Because XANES peaks with higher oxidation states have higher intensity, energy, and area, the average peak energy of the mixture is formed biased toward the peak of element with higher oxidation state.

To prove this, we designed the following experiment. XANES was measured by varying the ratio of pure IrO_2 and fully reduced metallic-Ir with similar particle size, as shown in Supplementary Note 5 Figure (a). The composition of the mixture was 40% IrO_2 and 60% metallic-Ir, and 70% IrO_2 and 30% metallic-Ir.

As the ratio of IrO_2 in the mixture increased, the area of the white line was directly proportional to the calibration curve (Supplementary Note 5 Figure (b)). However, white line peak energy of each mixture is further close to IrO_2 than each ratio, as shown in Supplementary Note 5 Figure (c). This suggests that electrocatalysts with higher oxidation state have a higher influence on the white line peak energy.

For our synthesized Ir-based catalysts, the particle size of each sample is same and metallic-Ir is located in core part. Thus, the white line area of Ir-based catalysts, which indicates amount of oxidized Ir species could roughly show thickness of the oxide. The white line peak energy of Ir-based catalysts, which highly affected by higher oxidation state elements, could roughly represents oxidation state of surface oxide layer.

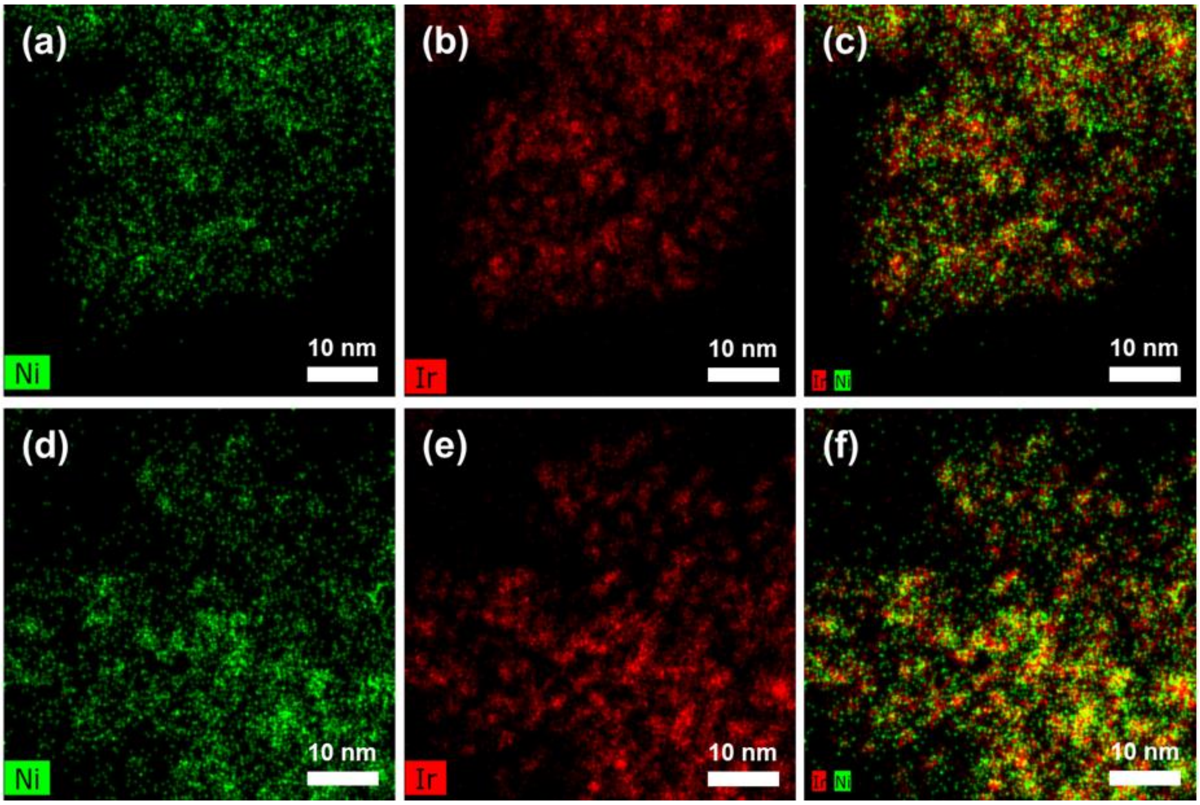

Supplementary Figure 1. STEM-EDX mapping images of Figure 1a and 1d. IrNi nanoparticles supported on carbon with different heat-treatment temperature of 400 °C and 1000 °C, which are denoted to IrNi/C-LT (a ~ c) and IrNi/C-HT (d ~ f), respectively. (a) Ni, (b) Ir, and (c) overall of Ni and Ir of IrNi/C-LT. (d) Ni, (e) Ir, and (f) overall of Ni and Ir of IrNi/C-HT.

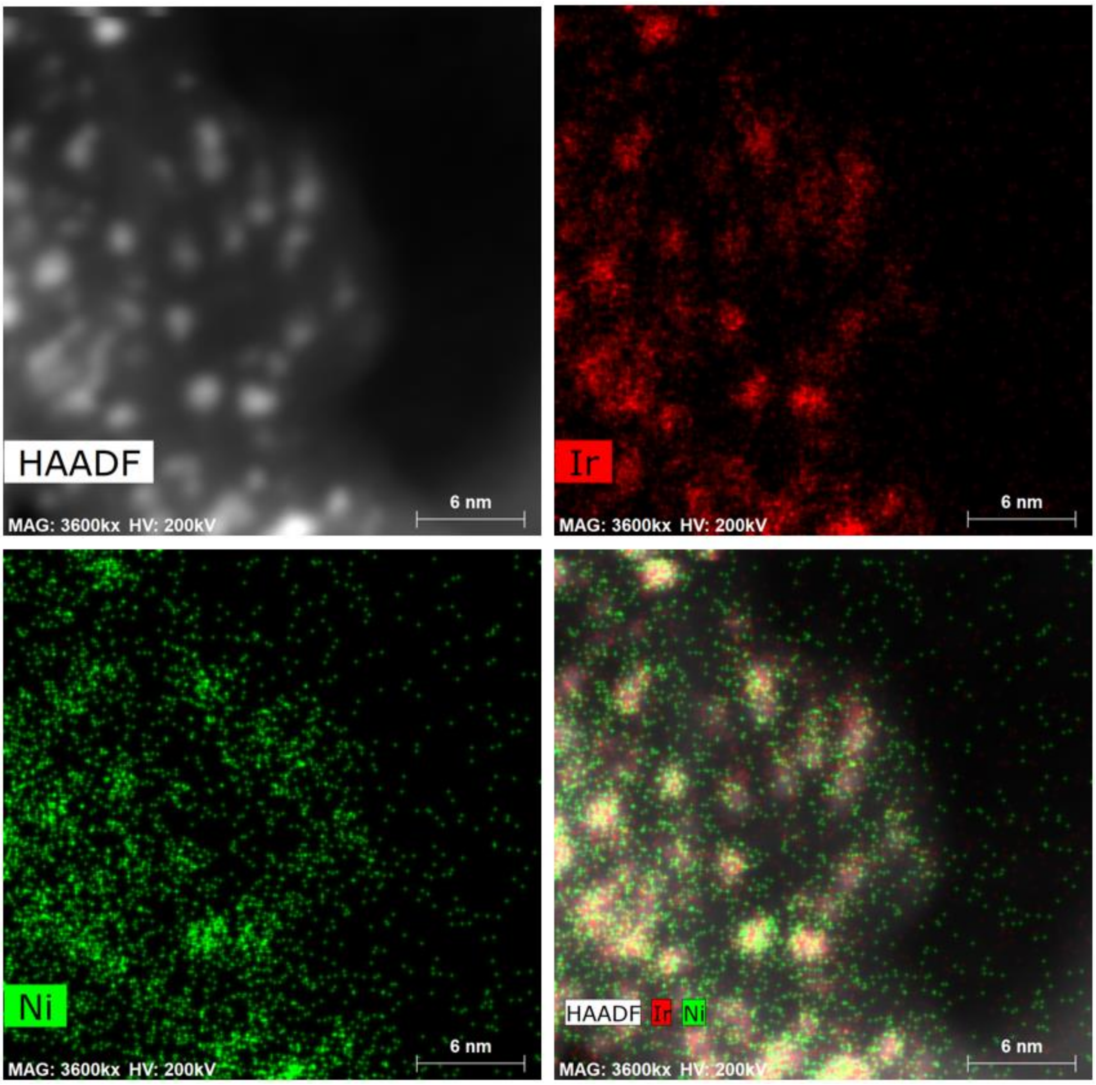

Supplementary Figure 2. STEM-EDX mapping images of IrNi/C-LT.

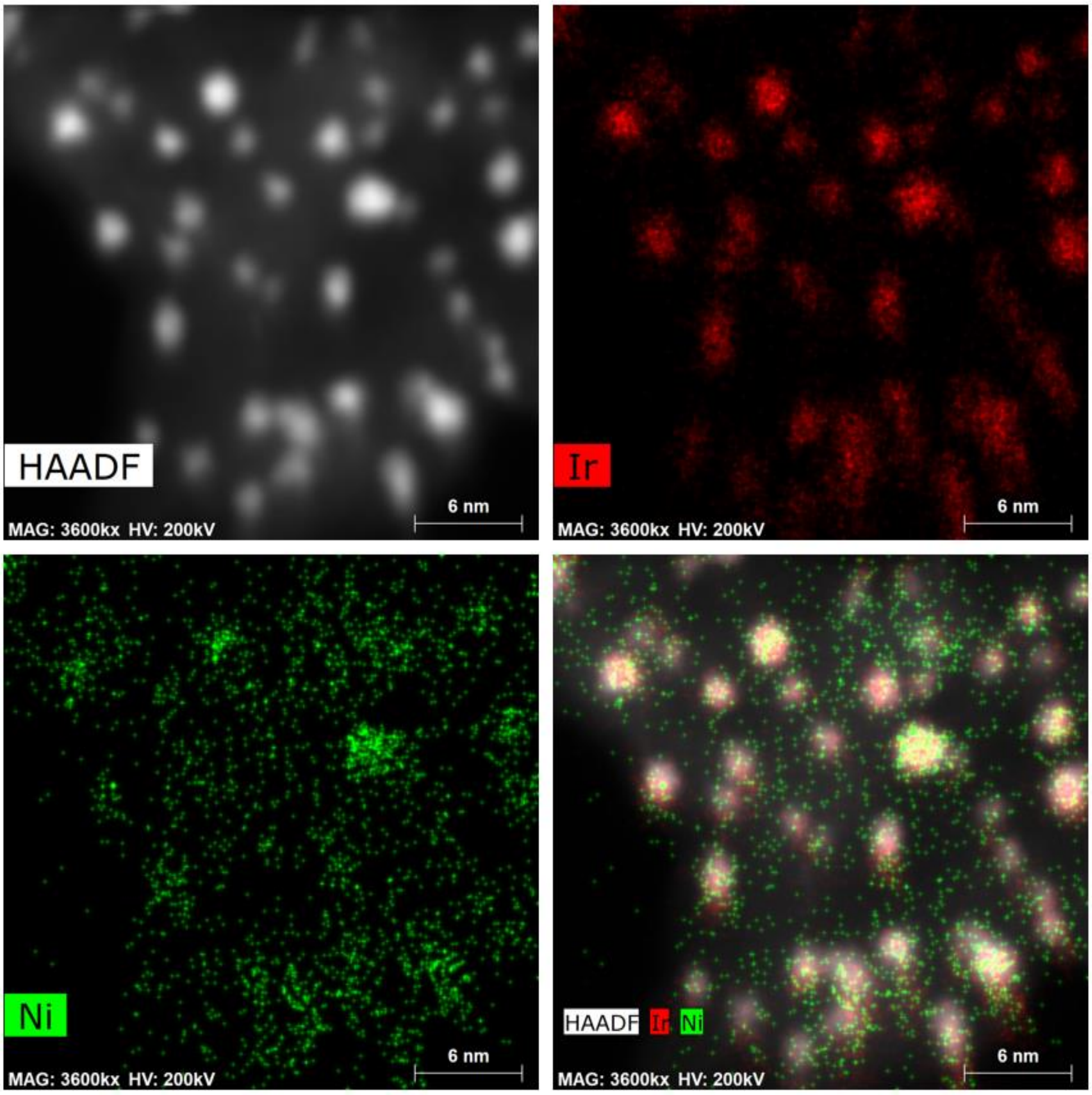

Supplementary Figure 3. STEM-EDX mapping images of IrNi/C-HT.

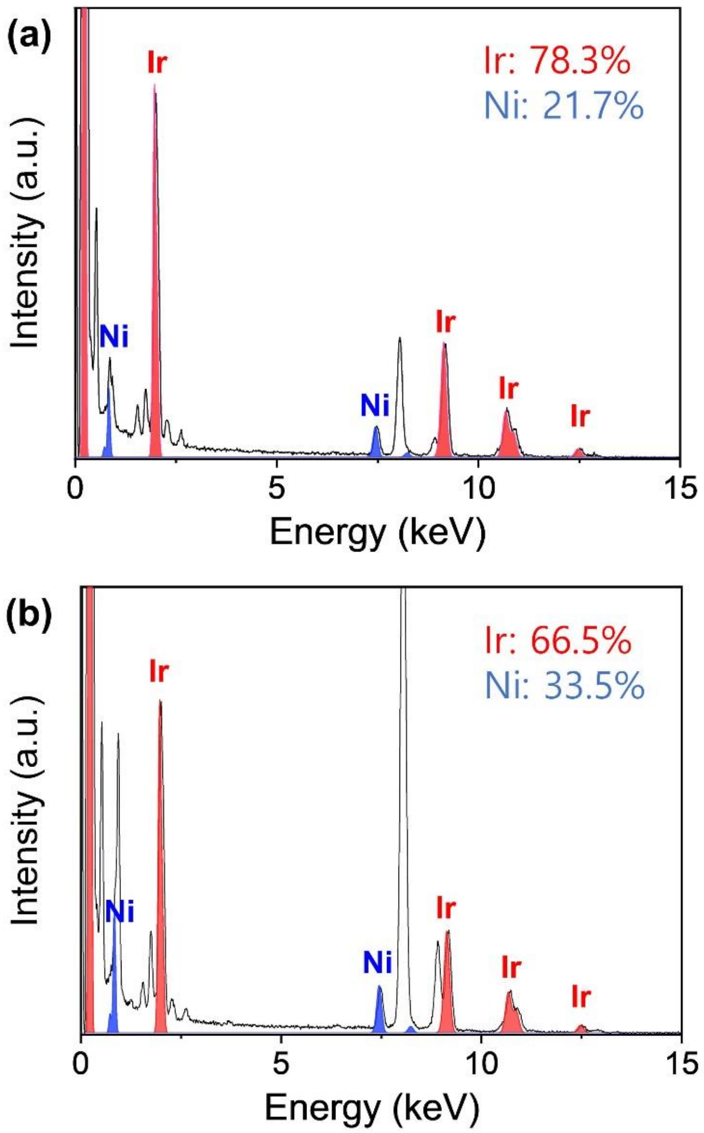

Supplementary Figure 4. EDX profile in TEM measurements of (a) IrNi/C-LT and (b) IrNi/C-HT.

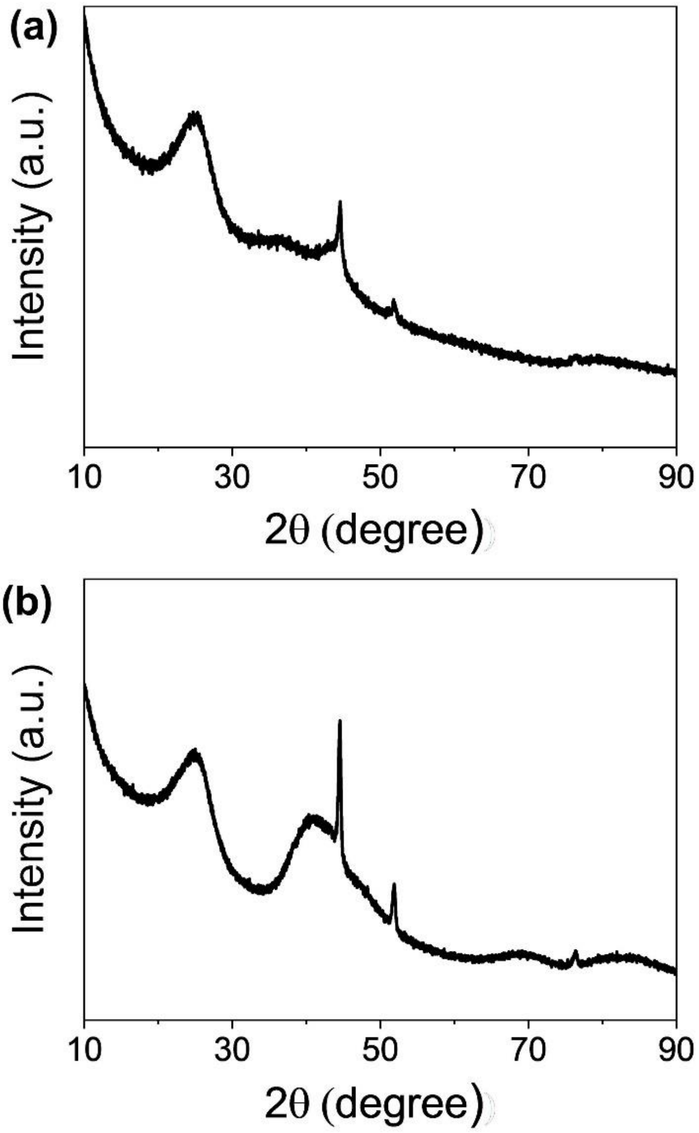

Supplementary Figure 5. Powder XRD patterns of (a) IrNi/C-LT and (b) IrNi/C-HT.

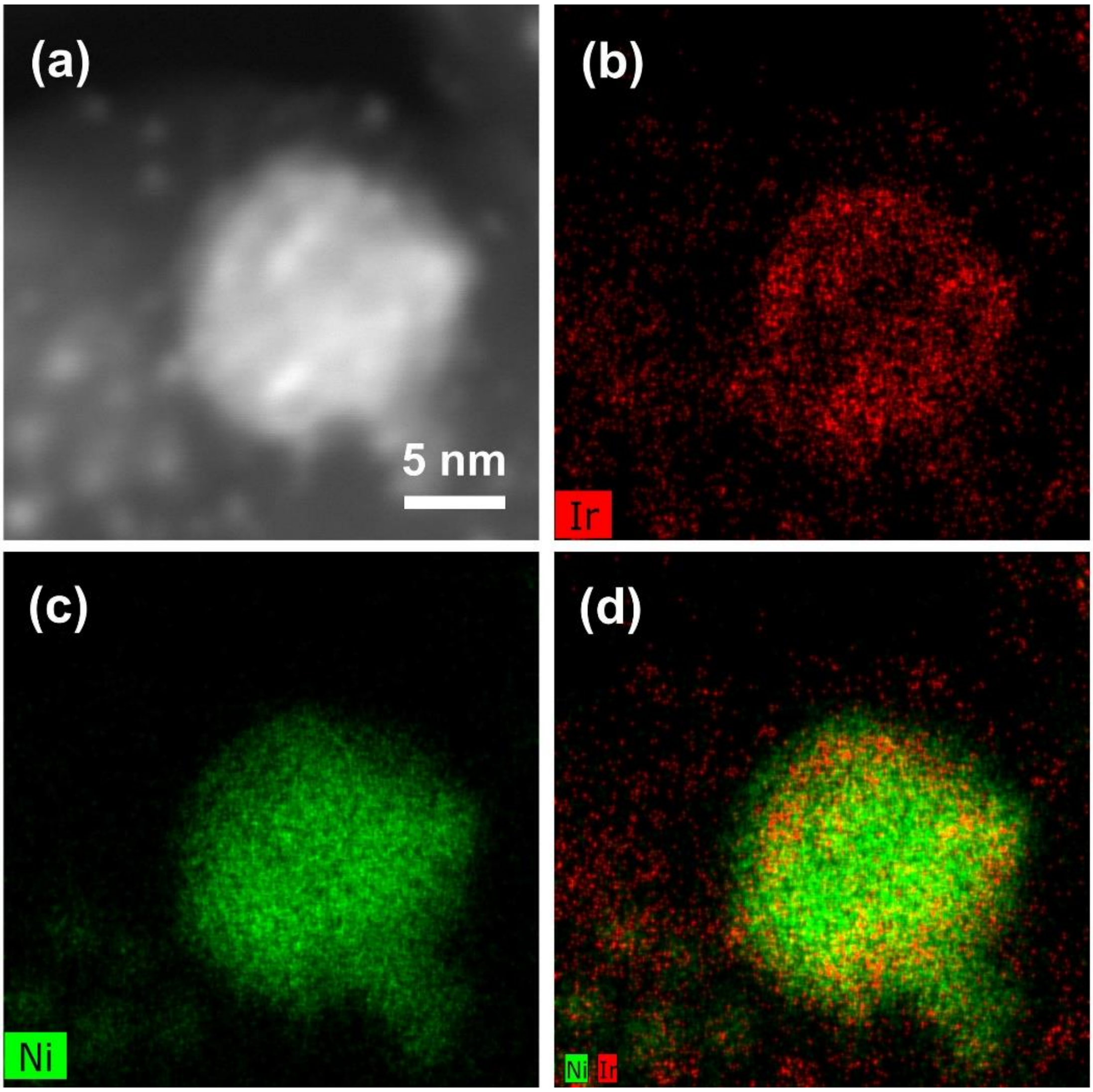

Supplementary Figure 6. (a) HAADF STEM image of IrNi/C-LT. STEM-EDX elemental mapping of large Ni nanoparticle in IrNi/C, showing the distribution of (b) Ir, (c) Ni, and (d) overall of Ir and Ni.

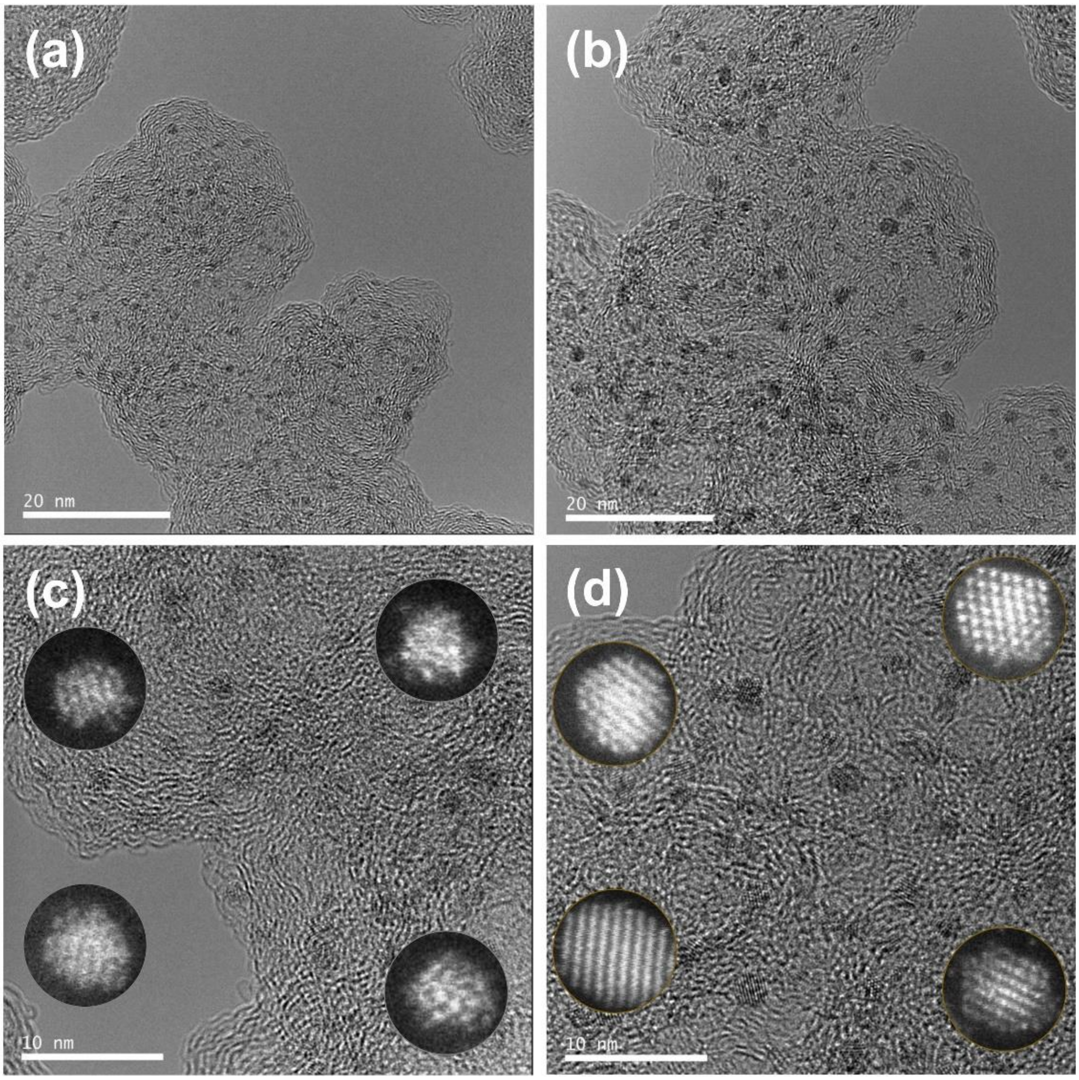

Supplementary Figure 7. HR-TEM images of Ir/C-LT and Ir/C-HT. (a, b) Low- and (c, d) high-magnification

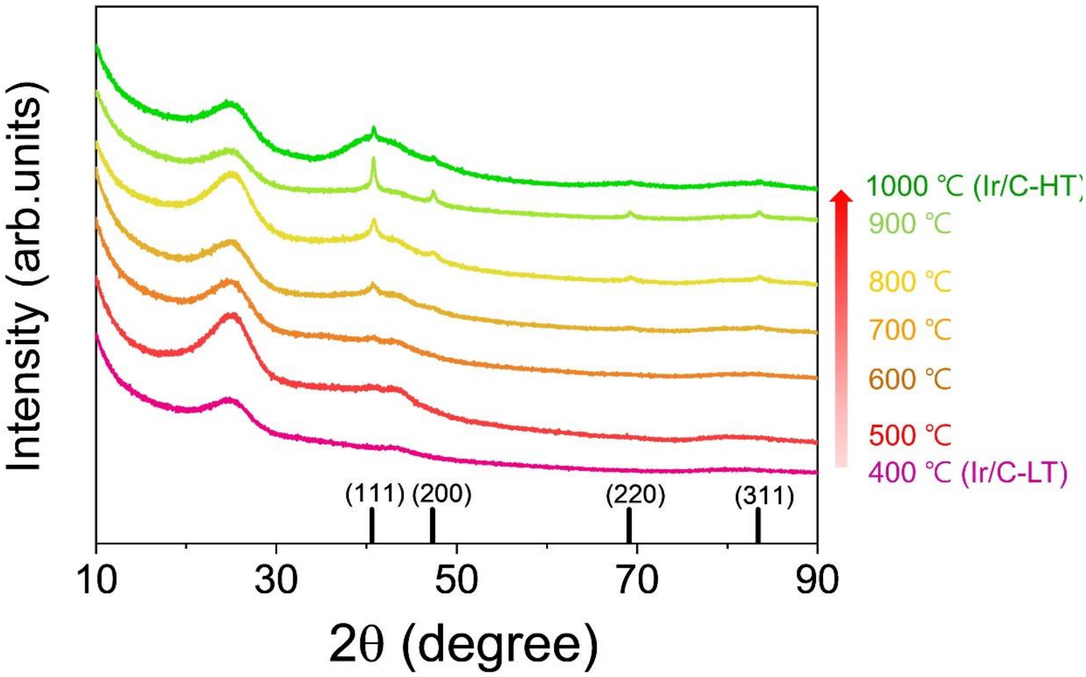

Supplementary Figure 8. XRD patterns of Ir/C with different synthesized temperature from 400 ℃ and 1000 ℃.

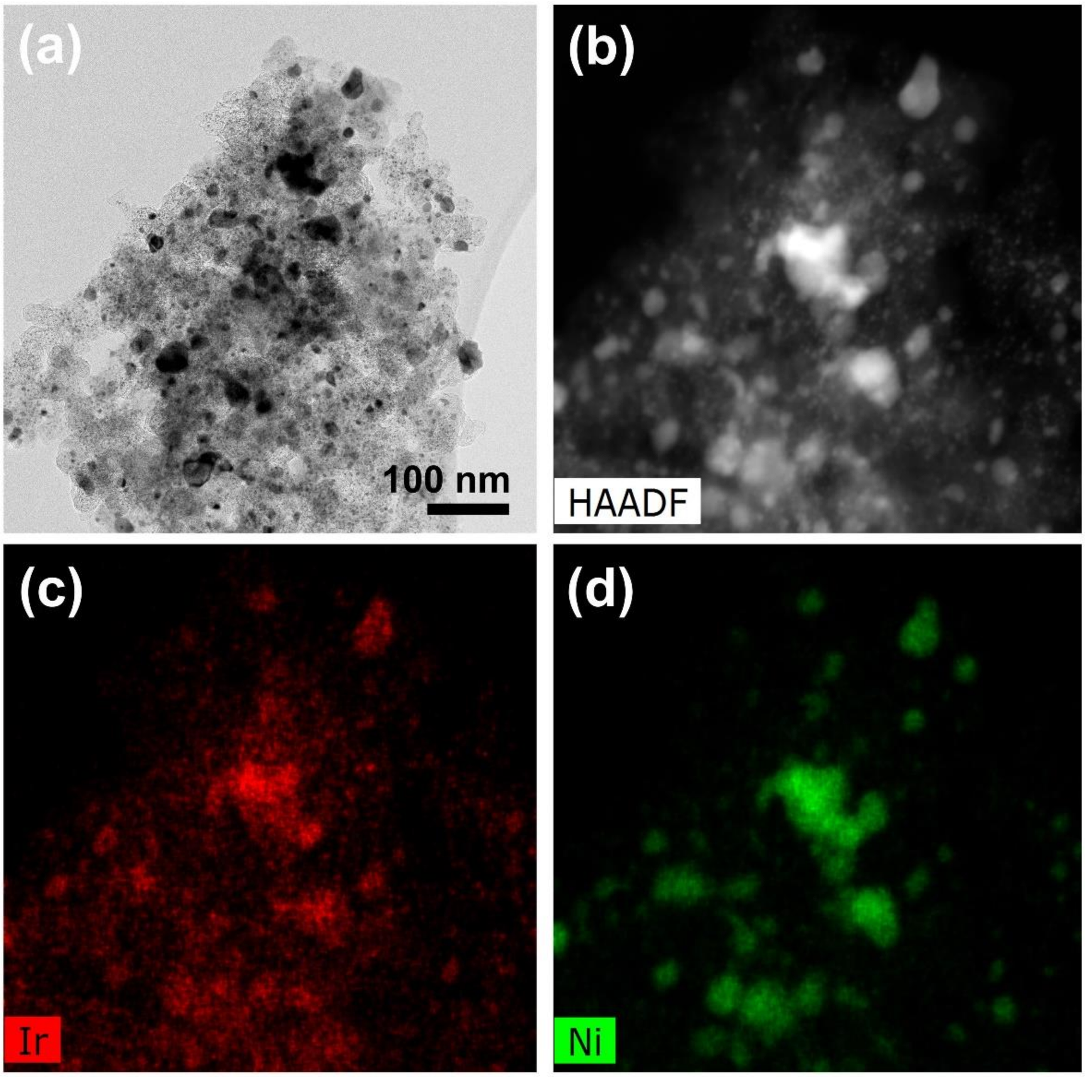

Supplementary Figure 9. (a) HR-TEM image of IrNi/C with heat-treatment at 1000 ℃. The heat-treatment gas condition was 10% H_2 (99.999%) and 90% N_2 (99.999%) from the beginning. (b) HAADF-STEM image and EDX elemental mapping of (c) Ir and (d) Ni in IrNi/C with heat-treatment at 1000 ℃ in 10% H_2 and 90% N_2.

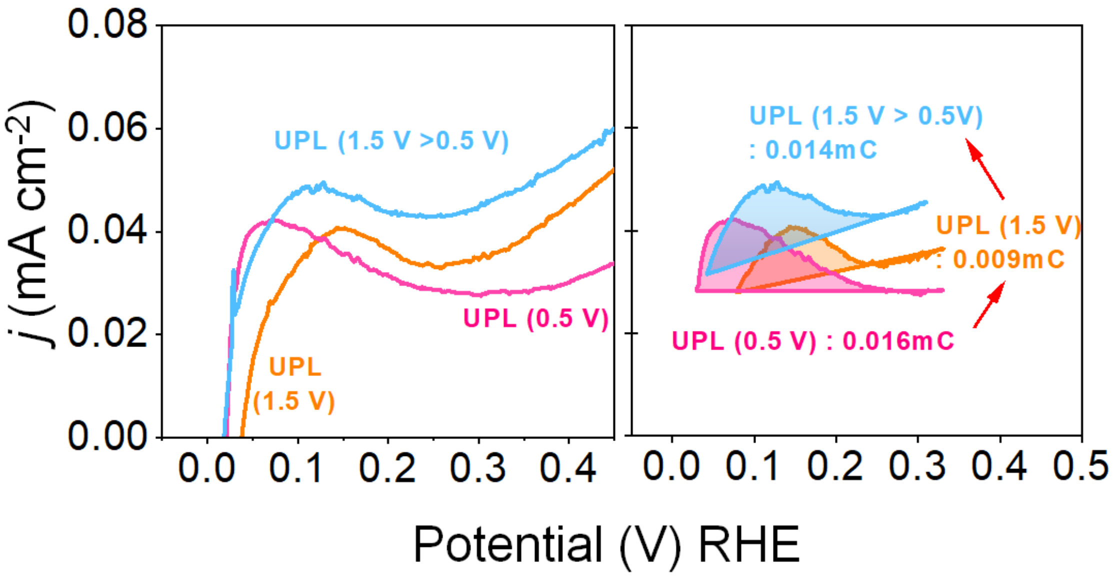

Supplementary Figure 10. Magnified graph of Figure 2b to show conversion of hydrogen adsorption-desorption (H_{upd}) peak.

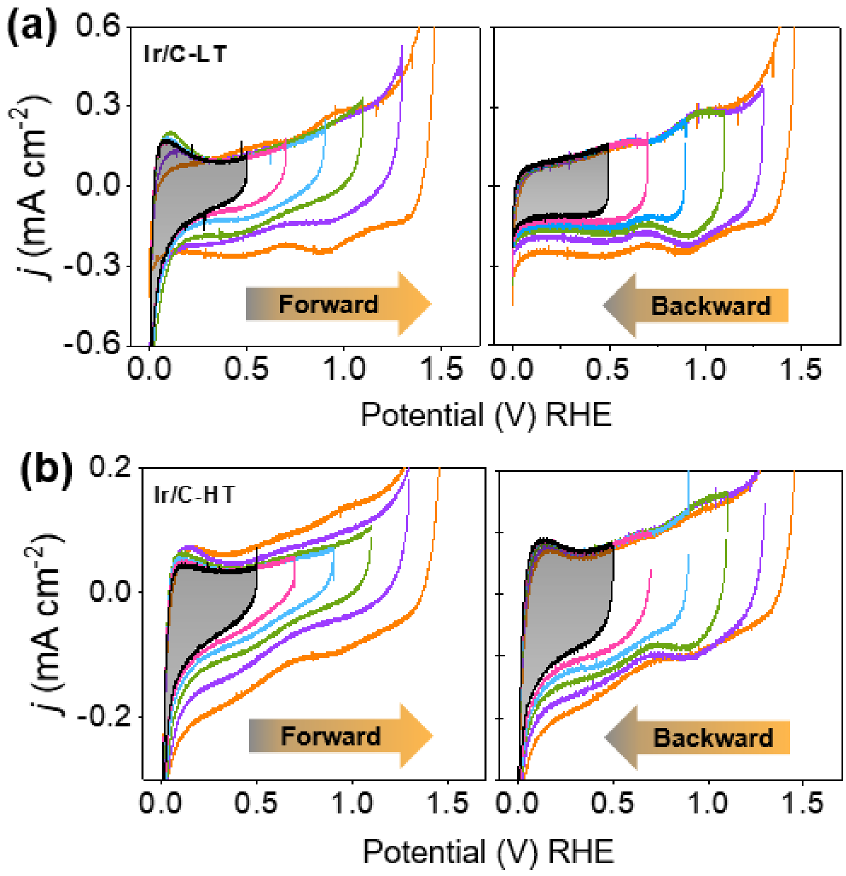

Supplementary Figure 11. Electrochemical property and catalytic reversibility of the Ir-based electrocatalyst. Cyclic voltammetry (CV) of (a) Ir/C-LT and (b) Ir/C-HT catalyst recorded with 50 mV s^{-1} in 0.05 M H_2SO_4 solution. The upper turning potential was increased from 0.5 to 1.5 V and decreased from 1.5 to 0.5 V in 200 mV steps.

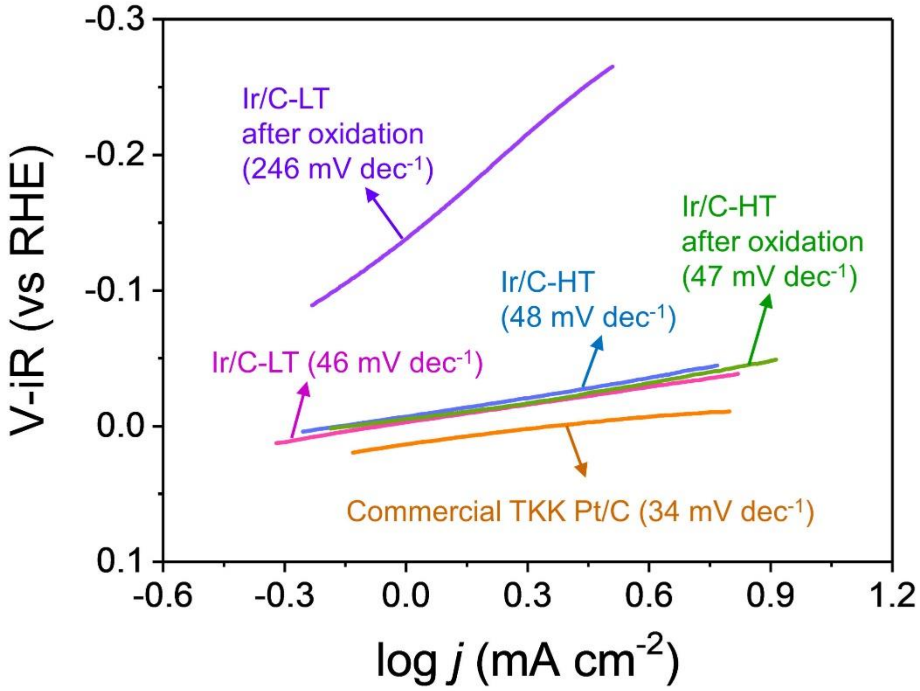

Supplementary Figure 12. Tafel plot of Ir/C-LT and Ir/C-HT for HER and HER after OER measurements. For comparison, the Tafel plot of commercial TKK Pt/C for HER is presented.

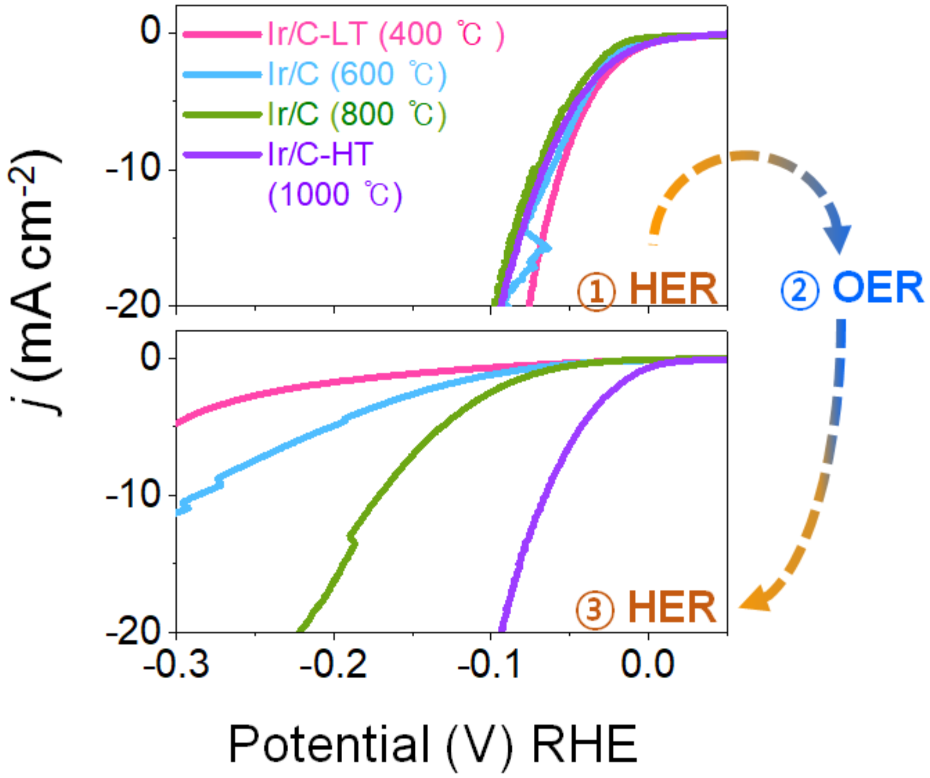

Supplementary Figure 13. iR-corrected current-potential curves of Ir/C with different synthesized temperature for HER and HER after OER in 0.05 M H_2SO_4 solution.

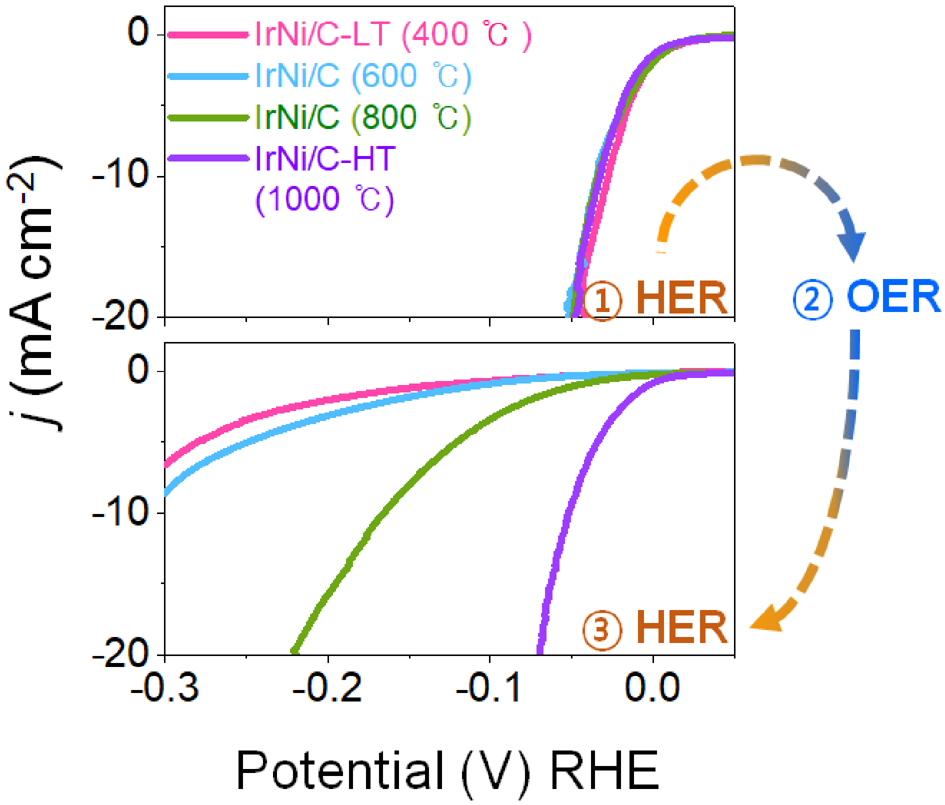

Supplementary Figure 14. iR-corrected current-potential curves of IrNi/C with different synthesized temperature for HER and HER after OER in 0.05 M H_2SO_4 solution.

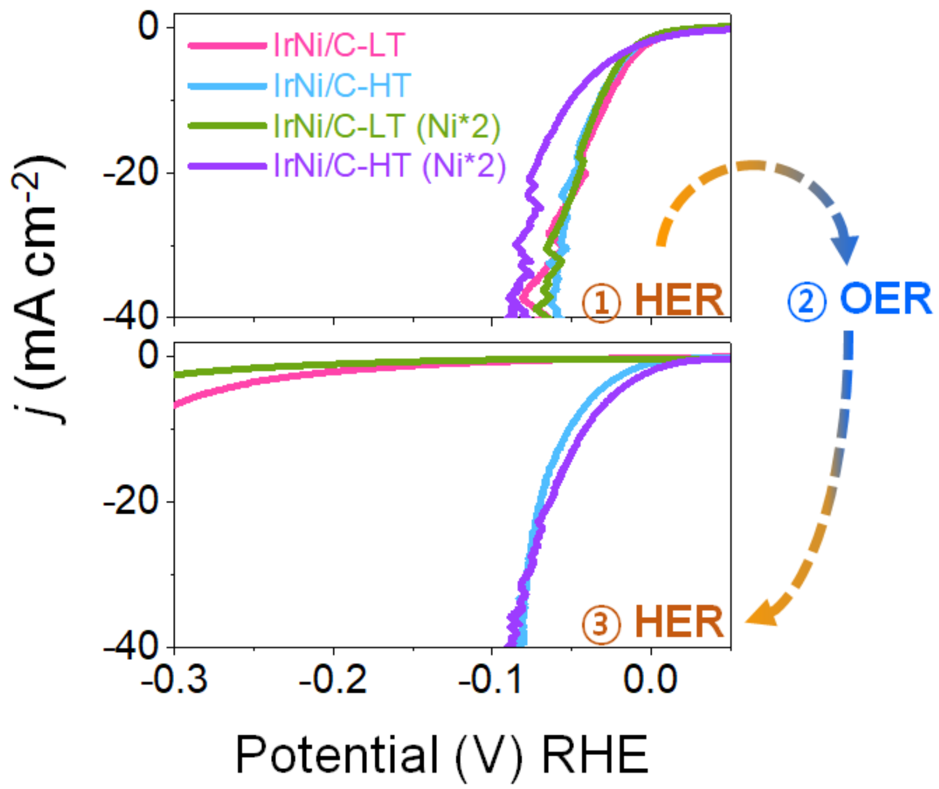

Supplementary Figure 15. iR-corrected current-potential curves of IrNi/C with different amount of Ni (based on input amount of Ni for manufacturing) for HER and HER after OER in 0.05 M H_2SO_4 solution.

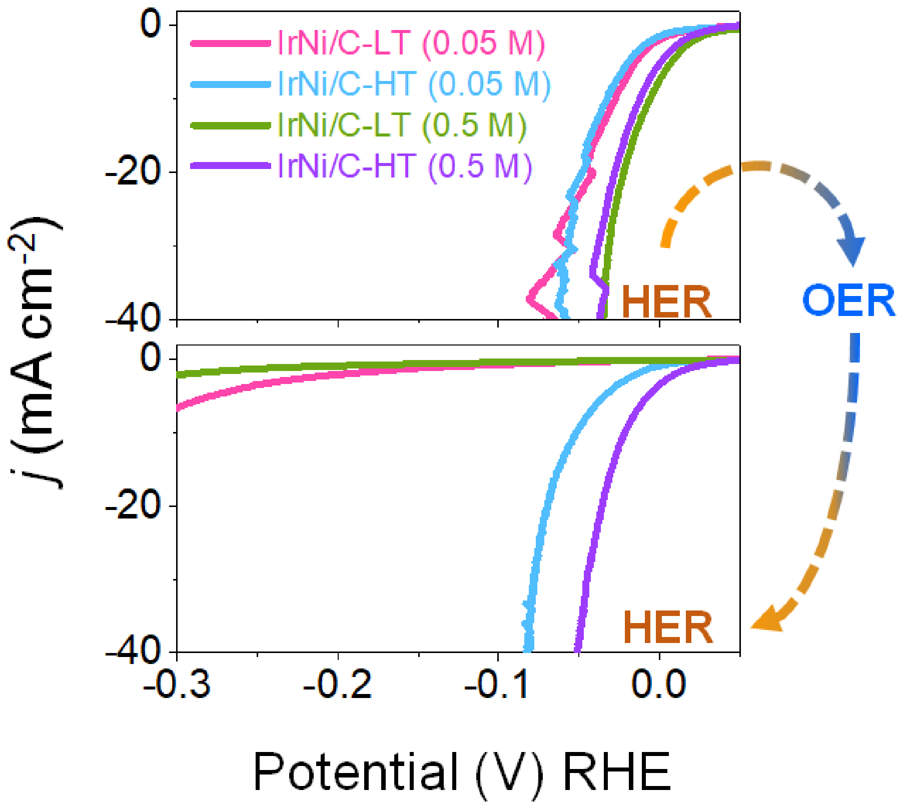

Supplementary Figure 16. iR-corrected current-potential curves of IrNi/C) for HER and HER after OER in 0.05 and 0.5 M H_2SO_4 solution.

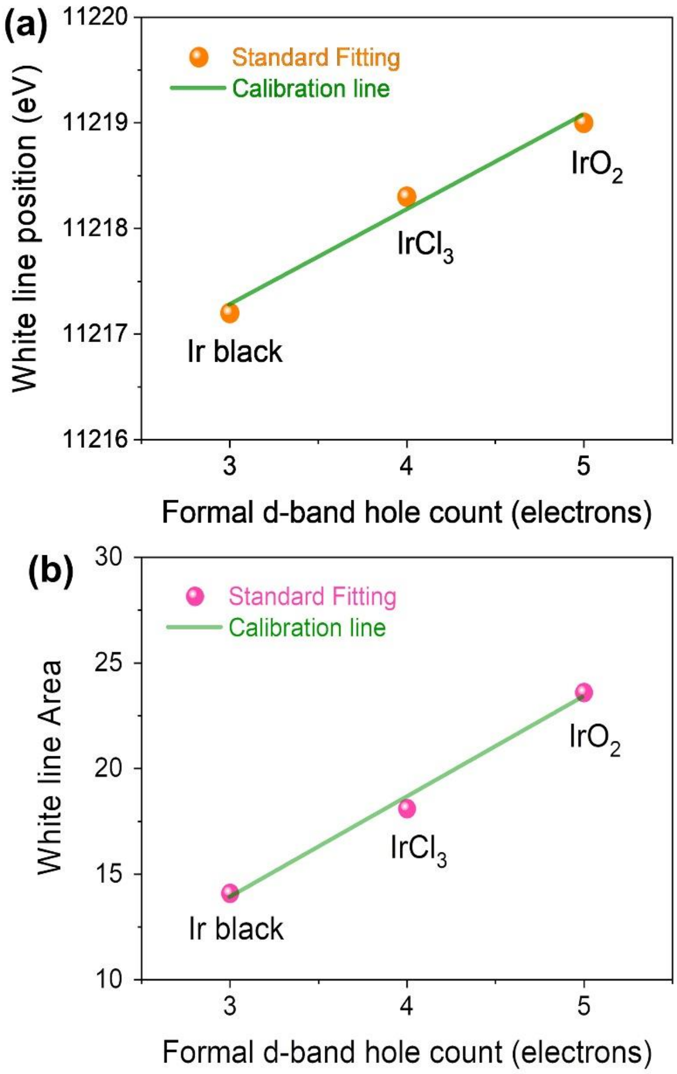

Supplementary Figure 17. (a) White line energy and (b) white line area of standard samples, such as metallic-Ir, $IrCl_3$ and IrO_2, as a function of the formal *d*-band hole count.

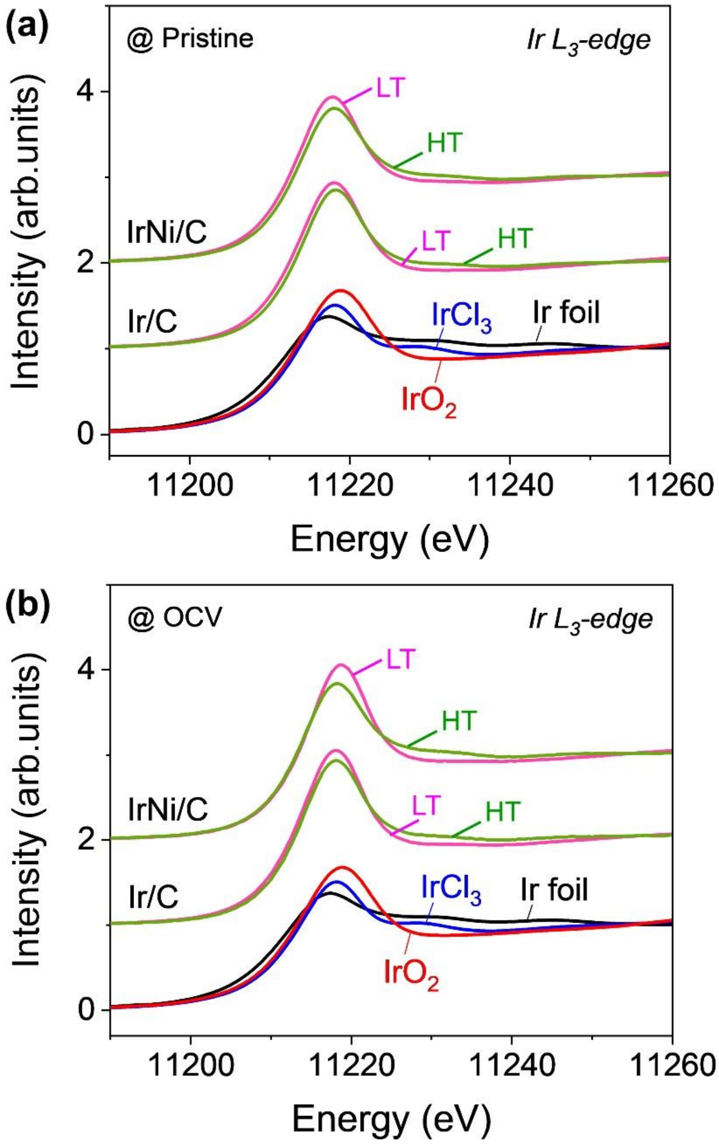

Supplementary Figure 18. Ir L_3-edge XANES spectra of (a) Ir/C-LT, (b) Ir/C-HT, (c) IrNi/C-LT and (d) IrNi/C-HT under pristine, OCV, OER and HER after OER condition. OER and HER were performed under chronoamperometry at 1.5 and –0.2 V_{RHE}, respectively.

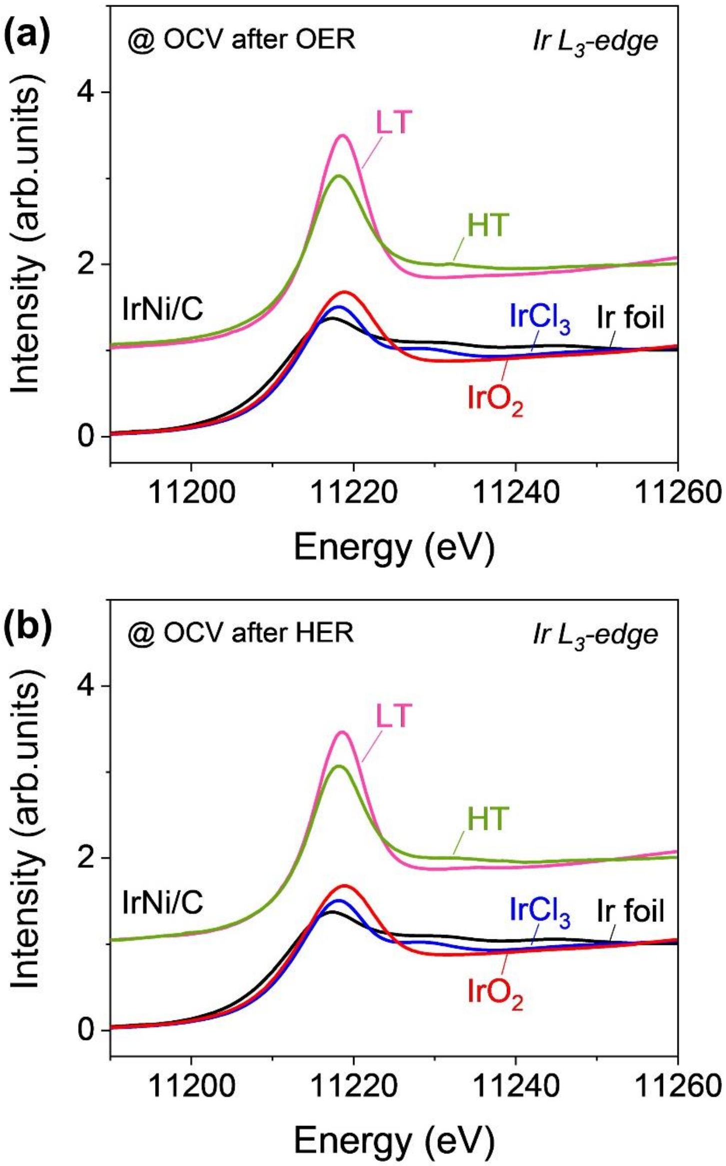

Supplementary Figure 19. Ir L_3-edge XANES spectra of IrNi/C-LT and IrNi/C-HT at (a) OCV after OER and (b) OCV after HER. OER and HER were performed under chronoamperometry at 1.5 and –0.2 V_{RHE}, respectively.

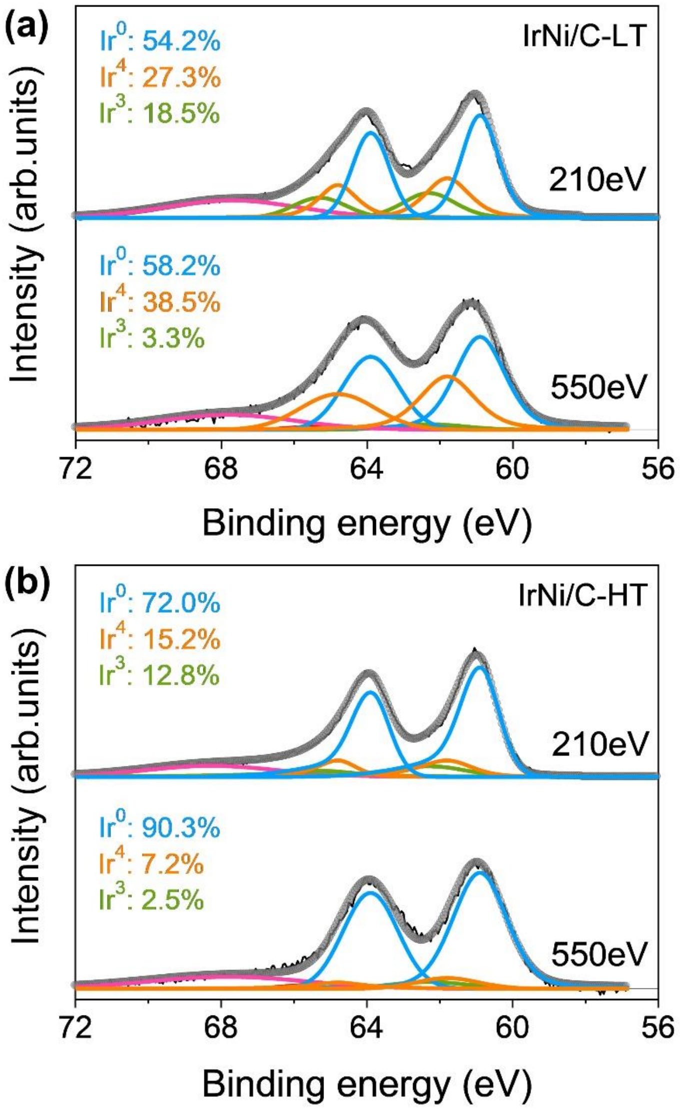

Supplementary Figure 20. Surface electronic structure of Ir in pristine IrNi/C-LT and IrNi/C-HT. Depth-resolved Ir 4f spectrum of (a) IrNi/C-LT and (b) IrNi/C-HT in spray coated electrode condition with photoelectron kinetic energies of 210 and 550 eV. The measured electrodes were prepared by spray coating the IrNi/C-LT and -HT electrocatalysts on carbon paper (Sigracet SGL 39BC). Depth-resolved XPS was measured at the 4D beamline of Pohang Accelerator Laboratory (PAL), Pohang, South Korea.

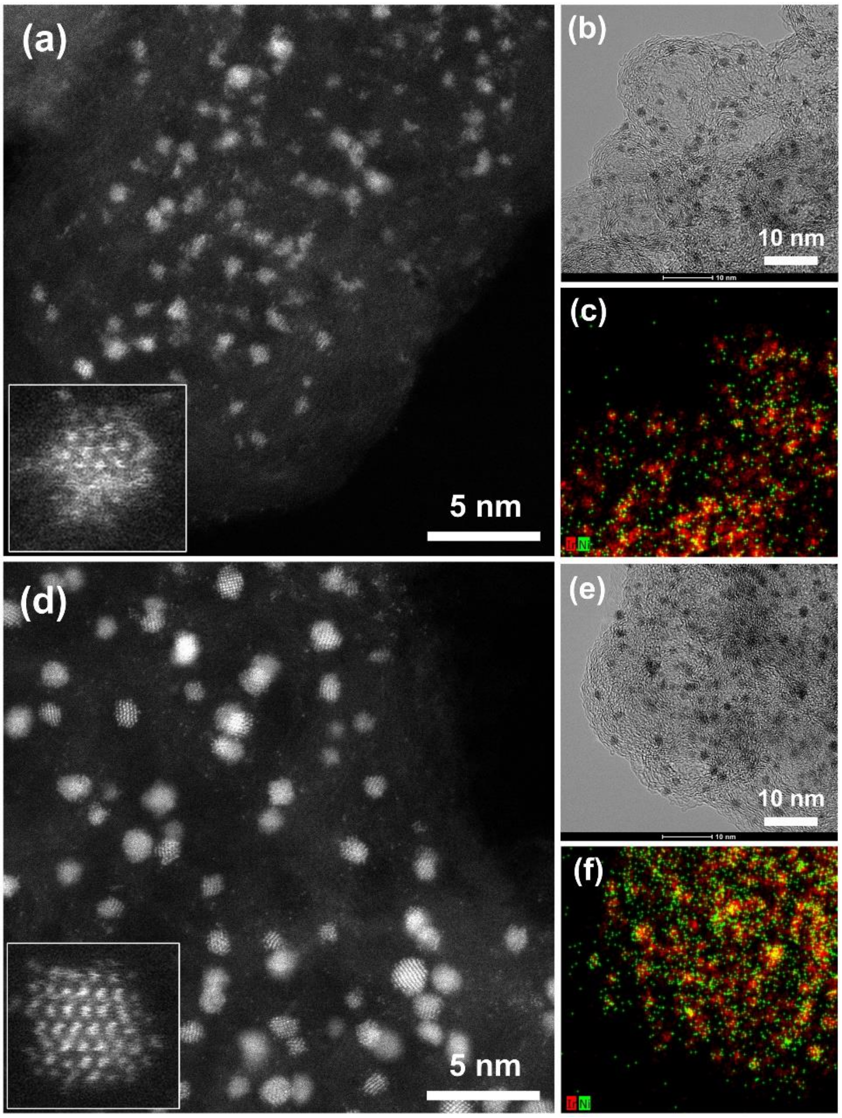

Supplementary Figure 21. Morphological of IrNi/C-LT and IrNi/C-HT after OER. (a) HADDF image, (b) TEM image, (c) STEM-EDX mappings of IrNi/C-LT after OER. (d) HADDF image, (e) TEM image, (f) STEM-EDX mappings of IrNi/C-HT after OER.

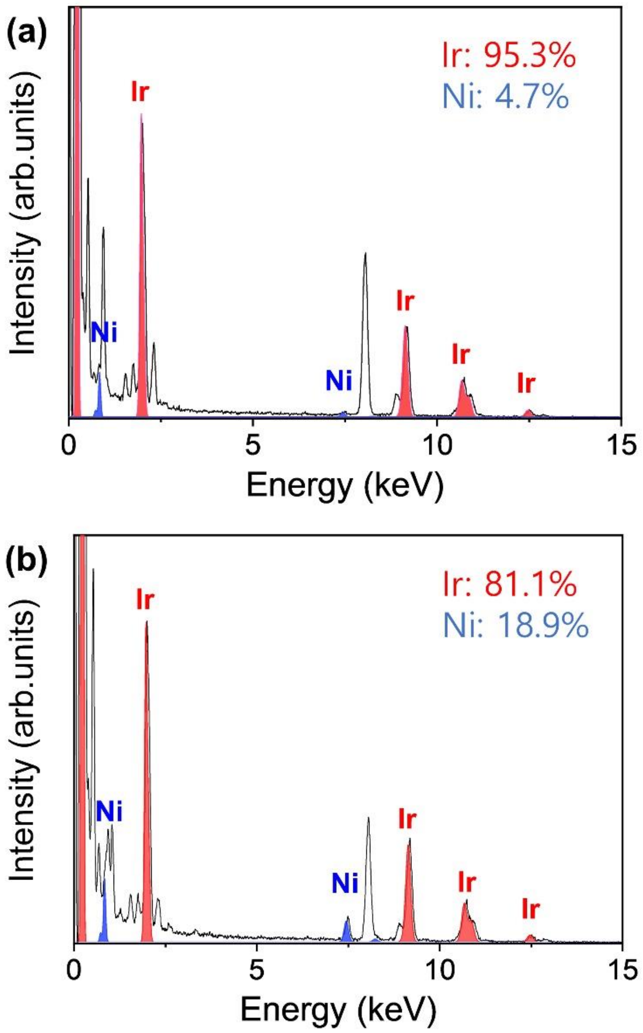

Supplementary Figure 22. EDX profile of IrNi/C-LT and IrNi/C-HT after OER measurement.

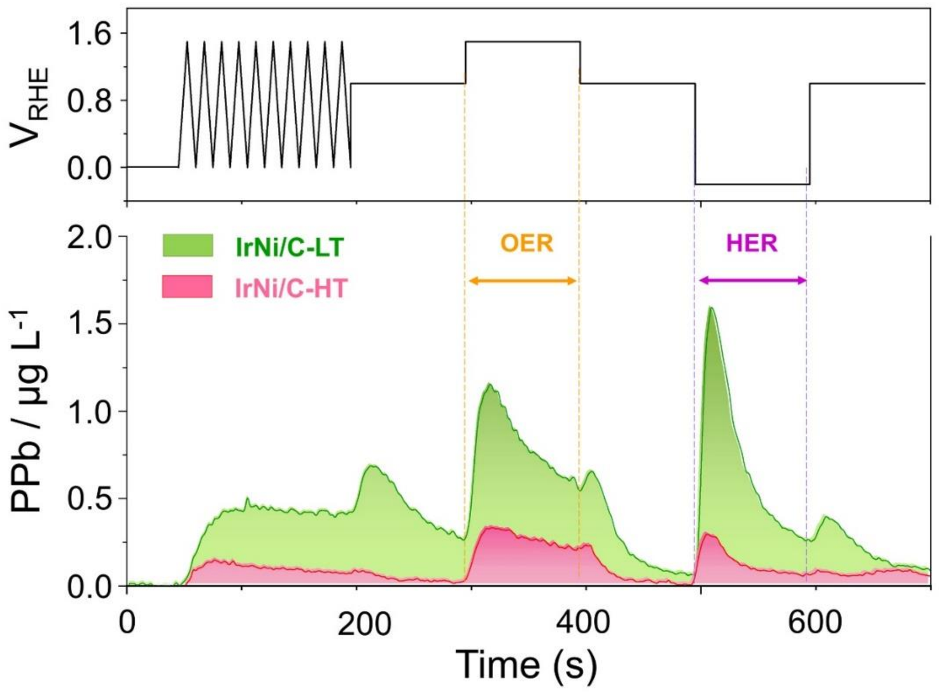

Supplementary Figure 23. Real-time Ir dissolution profile of IrNi/C-LT and IrNi/C-HT under representative experiment sequence. OER and HER were performed under chronoamperometry at 1.5 and –0.2 V_{RHE} for 100 s, respectively.

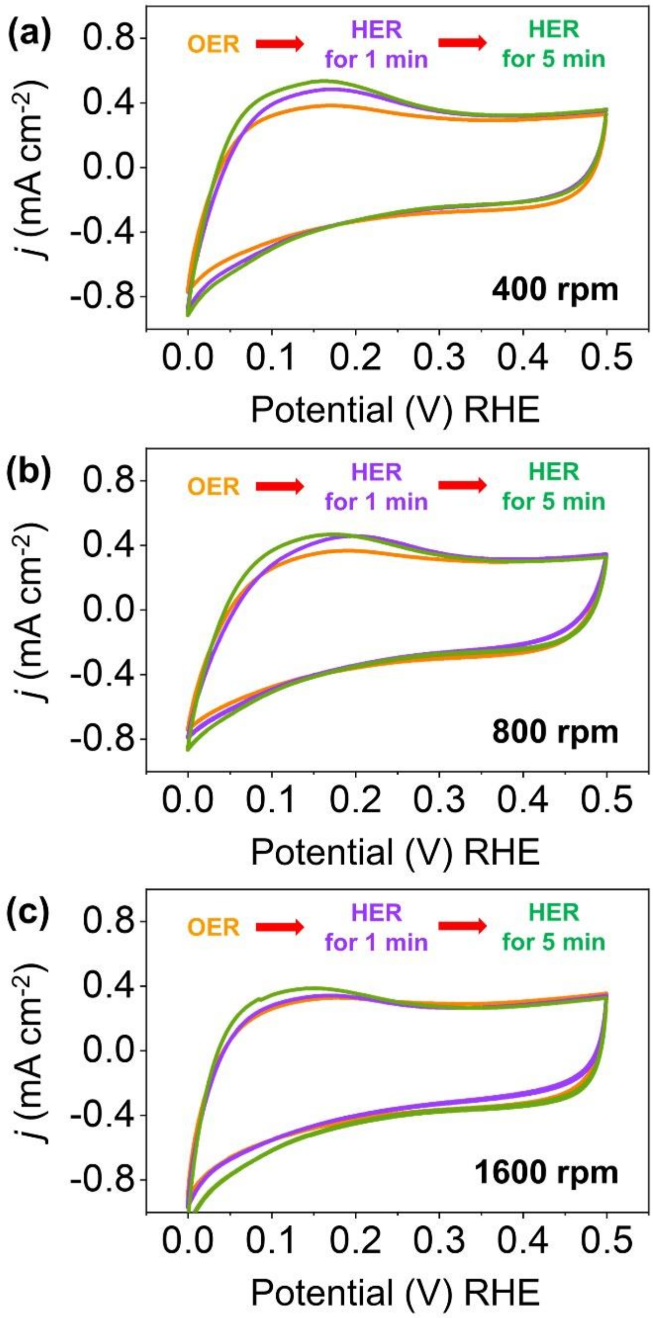

Supplementary Figure 24. Changes in the hydrogen adsorption-desorption (Hupd) peak of IrNi/C-HT after OER test, HER test for 1 min and HER test for 5 min (@ –0.2 V_{RHE}) with different rotation rate of RDE in 0.05 M H_2SO_4 solution.

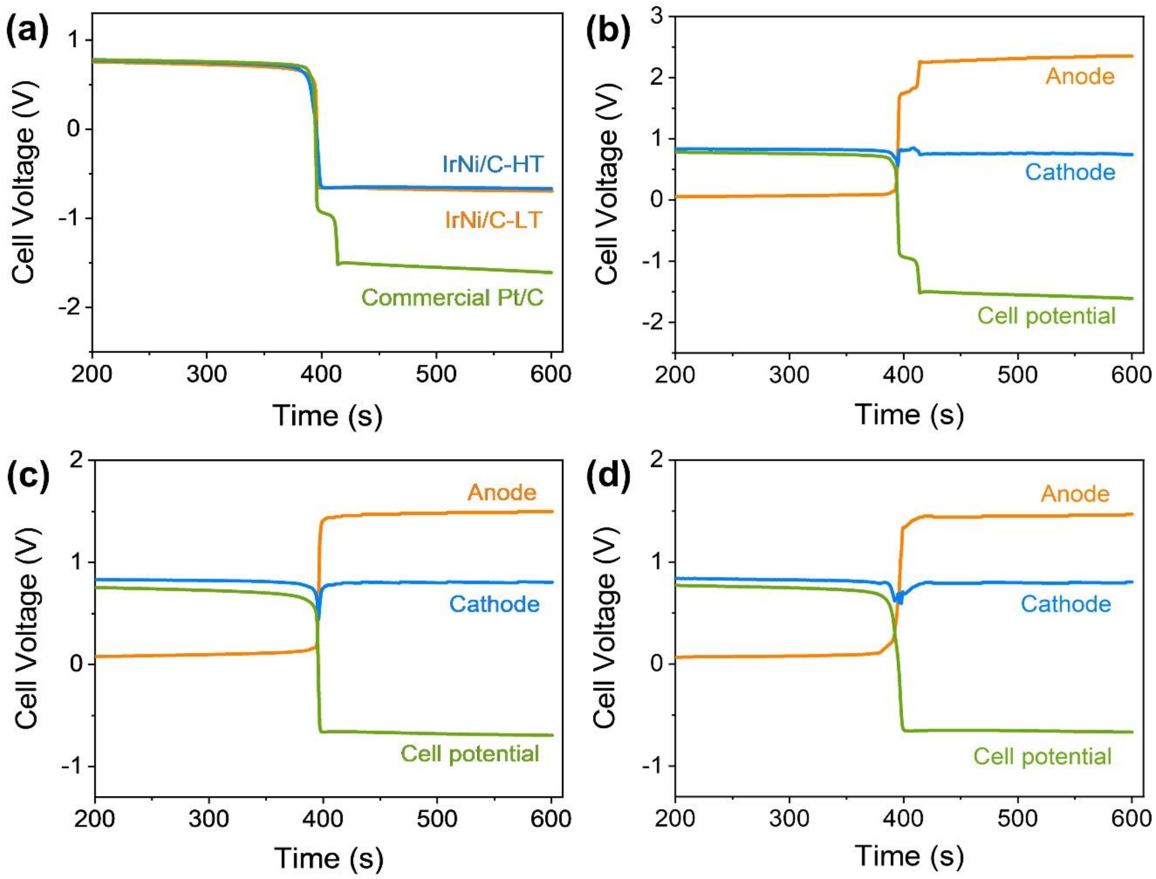

Supplementary Figure 25. Voltage reversal behavior of fuel cells. Cell voltage at fuel starvation as a function of voltage reversal time during the fuel starvation experiment. (a) Fuel cell potential behaviors of the commercial TKK Pt/C, IrNi/C-LT and IrNi/C-HT as the anode catalyst under the fuel starvation condition. The measured cell potentials of (b) commercial TKK Pt/C, (c) IrNi/C-LT and (d) IrNi/C-HT were divided to the anode and cathode potentials. Commercial TKK 46% Pt/C was used as a cathode catalyst for single cell measurement. For the fuel starvation condition, the gas on the anode side was changed from H_2 to Ar while maintaining the current density of 100 mA cm^{-2} for 600 s.

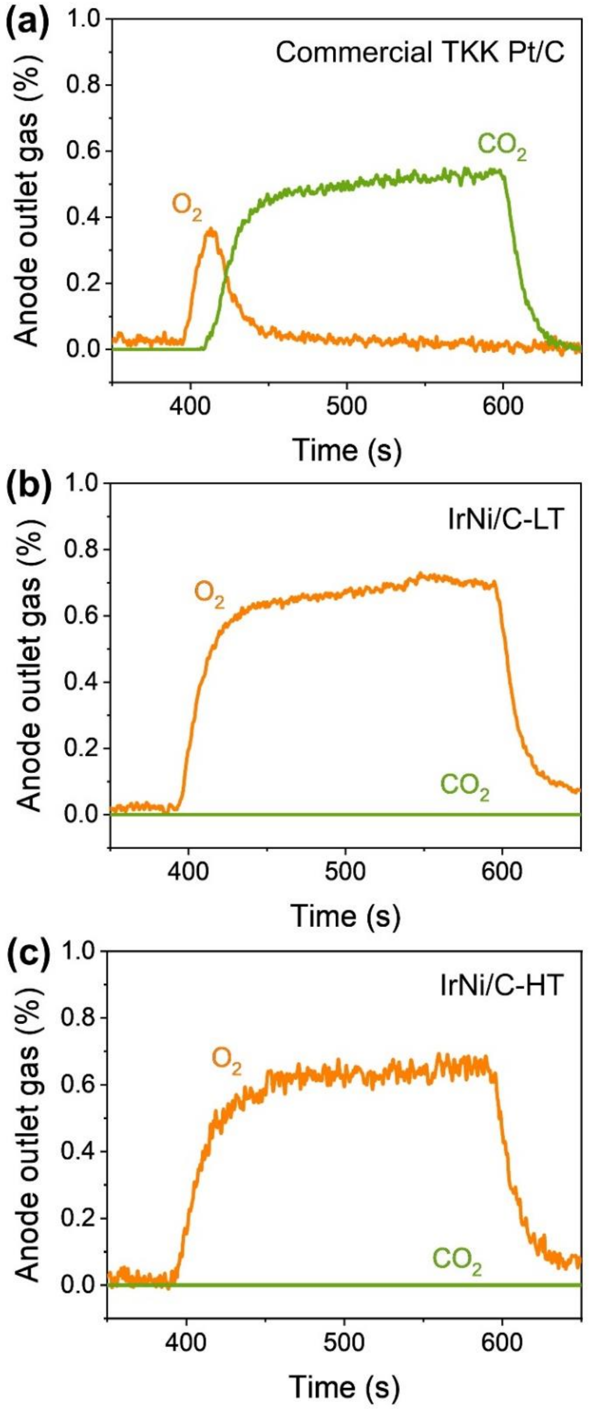

Supplementary Figure 26. *In situ/operando* gas chromatography-mass spectrometry (GC-MS) analysis on the exhaust gas from the anode of (a) commercial TKK 46% Pt/C, (b) IrNi/C-LT and (c) IrNi/C-HT during the fuel starvation condition. For the fuel starvation condition, the gas on the anode side was changed from H_2 to Ar at 400 s while maintaining the current density of 100 mA cm^{-2}.

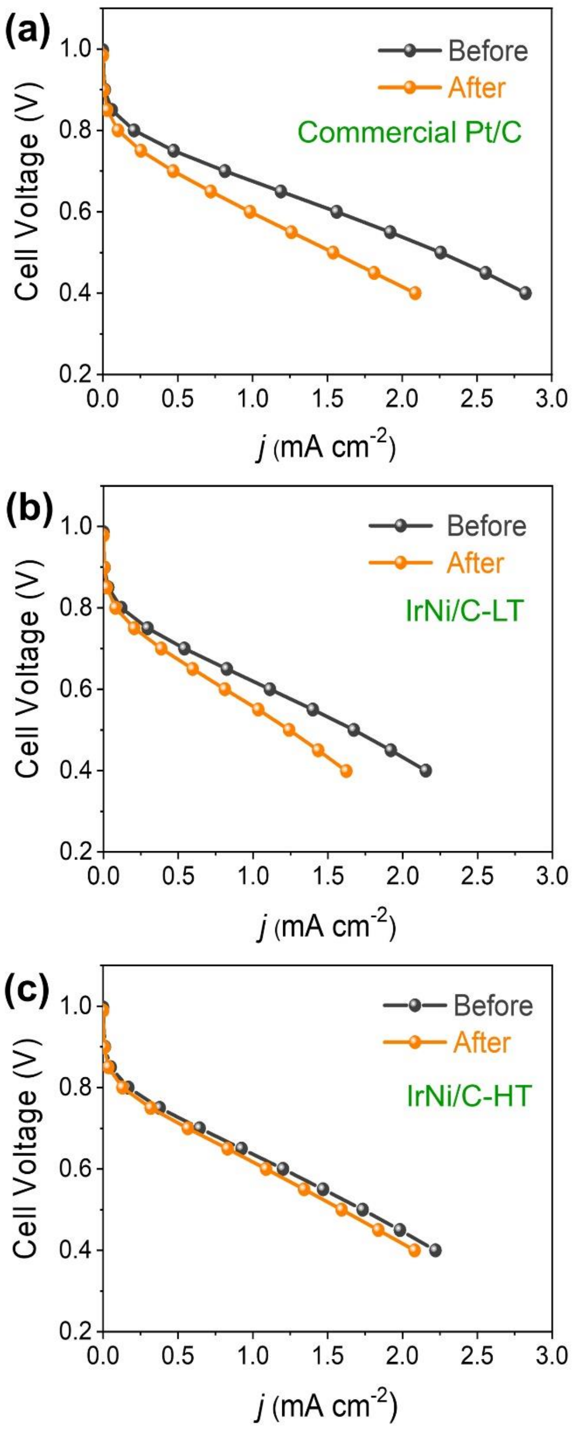

Supplementary Figure 27. Fuel cell polarization curves of (a) commercial TKK 46% Pt/C, (b) IrNi/C-LT and (c) IrNi/C-HT before and after the fuel starvation test.

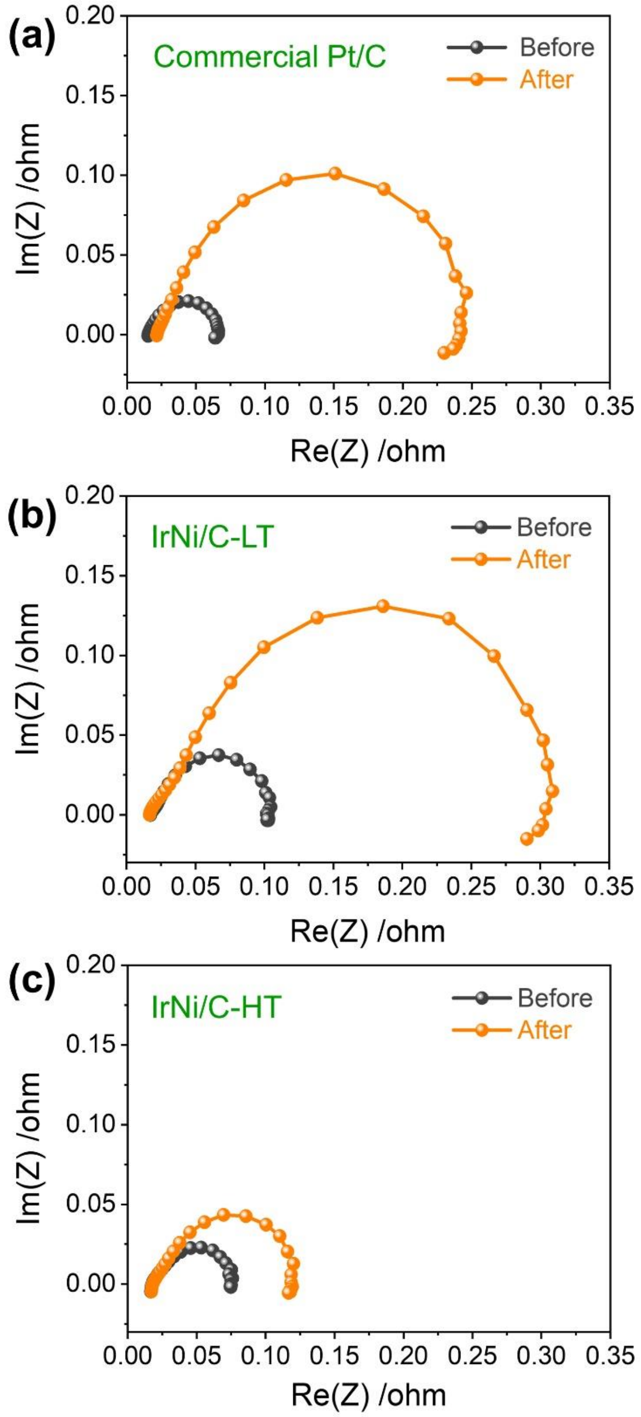

Supplementary Figure 28. Fuel cell impedance of (a) commercial TKK 46% Pt/C, (b) IrNi/C-LT and (c) IrNi/C-HT before and after the fuel starvation test.

Supplementary Table 1. Summary of deconvoluted depth-resolving XPS results.

Condition	IrNi/C-LT						IrNi/C-HT					
	210 eV			550 eV			210 eV			550 eV		
	Ir^{0}	Ir^{IV}	Ir^{III}	Ir^{0}	Ir^{IV}	Ir^{III}	Ir^{0}	Ir^{IV}	Ir^{III}	Ir^{0}	Ir^{IV}	Ir^{III}
Pristine	54.2	27.3	18.5	58.2	38.5	3.3	72.0	15.2	12.8	90.3	7.2	2.5
After OER	0	0	100	0	46.8	53.2	0	30.4	69.6	39.4	26.8	33.8
After OER followed by HER	0	0	100	0	67.2	32.2	38.2	46.2	15.6	57.8	31.7	10.5

Supplementary Table 2. Summary of changes before and after fuel starvation test.

Condition	MEA performance at 0.6V (A cm^{-2})		Membrane resistance (Ω)		Charge transfer resistance (Ω)		Mass data O_2 / CO_2 (cm^3)
	Before	After	Before	After	Before	After	*In situ* measure
TKK Pt/C 46%	1.56	0.98	0.0155	0.0215	0.0498	0.2191	0.53 / 3.98
	–37.2 %		+38.7 %		+339 %		
IrNi/C-LT	1.11	0.83	0.0174	0.0172	0.0850	0.2944	5.15 / 0
	–26.9 %		–1.2 %		+246 %		
IrNi/C-HT	1.20	1.09	0.0176	0.0176	0.0573	0.1014	5.04 / 0
	–9.2 %		0 %		+76.9 %		

References

1. Lee WH, *et al.* Carbon-Supported $IrCoO_x$ nanoparticles as an efficient and stable OER electrocatalyst for practicable CO_2 electrolysis. *Applied Catalysis B: Environmental*, 118820 (2020).
